# Stability Testing Reveals Photoselective Clipping of Heavy Chain C‑Terminal Amino Acids That Leads to Fragmentation and Aggregation of an Antibody Fab Fragment

**DOI:** 10.1021/acs.molpharmaceut.5c00592

**Published:** 2025-09-10

**Authors:** Arka Mukhopadhyay, Kersti Karu, Paul A. Dalby

**Affiliations:** † Department of Biochemical Engineering, 4919 University College London, Gower Street, London, WC1E 6BT, U.K.; ‡ Department of Chemistry, University College London, 20 Gordon Street, London, WC1H 0AJ, UK

**Keywords:** A33Fab, External stress, Photoexposure, Aggregation, Fragmentation

## Abstract

We built a custom device to subject an antibody fragment A33 Fab to controlled stress conditions that combined pH, temperature, agitation, and LED-based light exposure in polypropylene microplates; to simulate the real-world challenges it may encounter during storage and transportation and to evaluate the key degradation routes in Fab formulations. We also explored the addition of Tween 80 as a surfactant and the impact of plate surface siliconisation. Monomer loss and fragmentation was monitored by size-exclusion chromatography, aggregate formation determined by changes in hydrodynamic radius in DLS, and chemical modifications identified through intact mass analysis by LC-MS, and N-terminal sequencing. The findings indicated that the light exposure conditions often interacted with other factors. In particular, light exposure in the UV to blue range led to chemical degradations that led to greater susceptibility to aggregation, particularly at elevated temperatures. Interestingly, while Tween 80 provided stabilization, particularly within siliconized plates, the presence of Tween 80 also promoted losses due to light exposure, consistent with previous findings that Tween 80 could act as a photosensitizer. Exposure of A33 Fab to light led to sequential losses of amino acids selectively from only the heavy chain C-terminus, indicating a photosensitive hotspot in that region of the Fab structure. These also suggested that photoinduced clipping of the heavy chain C-terminus increased the susceptibility of A33 Fab to fragmentation into heavy and light chains and aggregation, consistent with previous work in which flexibility-suppressing mutations of the hinge region decreased the aggregation kinetics.

## Graphical abstract

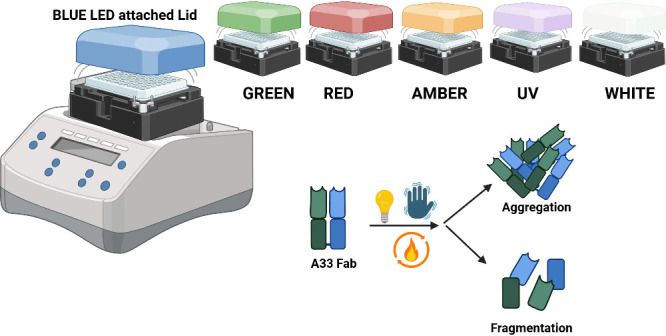

## Introduction

Ensuring the stability of biotherapeutics is critical for maintaining their safety and effectiveness.^1,2^ Stability, as per the guidelines set forth by the World Health Organization (WHO)^3^ on stability testing of pharmaceutical products (WHO, 1996), refers to the capacity to maintain the chemical, physical, microbiological, and biopharmaceutical characteristics of a drug molecule within predetermined parameters throughout its required shelf life. These guidelines also define the appropriate stability testing and accelerated stability testing that should be done to evaluate formulated pharmaceutical products.^4^ Various factors are known to impact protein stability, including temperature, pH, ionic strength, redox potential, exposure to light, interfacial surfaces, and shear, with many leading to aggregation or fragmentation.^5–7^ Aggregates can form either in solution or on surfaces through adsorption with the aggregation process being reversible or irreversible. The stability of proteins and consequently their aggregation behavior are influenced to varying degrees by the various stress factors during their production, processing, storage, transportation and administration to patients, such as through mechanical agitation stresses due to pumping and shaking, or from freeze–thawing.^5–7^

The intensity and duration of stress exposures influences various characteristics of resulting aggregates, including their size, concentration, solubility, charge, and morphology.^8^ Aggregation can arise from stresses encountered at interfaces between air and liquid phases,^6^ solid and liquid phases,^9,10^ and liquid–liquid phases.^11^ The loss of protein to aggregate formation during bioprocessing is thought to result from the combined effects of interface and shear stress, suggesting that shear stress alone is unlikely to cause protein aggregation.^9,12^

Protein solutions can endure two types of mechanical stress during transportation: low-*g*-force vibrations, like those from truck movements, and high-*g*-force shocks, such as dropping. High-*g*-force shocks have previously been considered the main culprits behind protein aggregation.^8^ However, a recent study found that micron-sized aggregates produced by low-*g*-force stresses from orbital shaking at 320 rpm bore closer resemblance to actual shipping-induced aggregates than those from high-*g*-force shocks,^1^ suggesting that low-*g*-force stresses dominated the aggregate formation during transportation.

Forced degradation investigations employing horizontal and orbital shakers are commonly utilized to assess stability against agitation stress. These have been calculated to produce accelerations below 2*g*, indicating low-*g*-force stresses.^8^ These stresses, such as vibrations during air or truck transport, are repetitive, with frequency and acceleration being characteristic parameters. The critical parameters for a microplate-based orbital shaker method were recently investigated for optimizing protein formulations. Analysis of monomer recovery with shaking speeds ranging from 0 to 3000 rpm, and varying liquid height, revealed a significant decrease in monomer content under higher shaking speed conditions in which the air–liquid interfacial area increased.^13^

Nonionic surfactants such as Tween 20 (TW20) and Tween 80 (TW80) are commonly utilized in protein formulations to prevent aggregation, particularly that caused by adsorption onto surfaces.^14,15^ While low concentrations are often sufficient to prevent the absorption of monoclonal antibody (mAb) to air–liquid interfaces, higher concentrations are often used where needed to prevent aggregation caused directly by protein–protein interactions, and has also been demonstrated to avert aggregation induced by mechanical agitation and freezing.^16^

Chemical modifications such as deamidation,^17^ or oxidation^5,17^ can potentially induce structural alterations in proteins, and could ultimately lead to protein aggregation, increased immunogenicity, and interfere with therapeutic function.^5^ Oxidation can arise through several mechanisms, influenced not only by redox potential but also by UV light exposure, metal contaminants, heat, and mechanical agitation. Moreover, excipients can contribute to oxidation. For example, TW20 and TW80 can undergo auto-oxidation or photo-oxidation, generating reactive peroxides that subsequently induce oxidative changes in proteins.^15,18,19^

Therapeutic proteins can also be sensitive to light and so their tolerance is typically tested under conditions set out in ICH Q1B guidelines, which includes 1.2 M lux h exposure to white light and near-ultraviolet (UV) spanning 320–400 nm (peak 350–370 nm). During typical laboratory and manufacturing processes, proteins are mainly exposed to visible light (>400 nm) and to a lesser extent to UV light (350–400 nm range)^20^ derived from various light sources including metal halide, fluorescent, and LED lamps. While monoclonal antibodies (mAbs) have few chromophores able to absorb visible light, their degradation upon exposure to visible light has been observed in the presence of photosensitizers. Such photoactive compounds can appear as impurities from mAb manufacturing, such as riboflavin from cell culture or trace iron from excipients,^21^ while polysorbates (Tween), commonly added during formulation, are an additional potential source of photoactive substances.^19^ These nonproteinogenic photosensitizers can absorb visible light and induce protein degradation through photo-oxidation mechanisms, leading to the formation of activated oxygen species that oxidize susceptible amino acids like Trp, His, Met, Tyr, and Cys.^19^ Increased concentrations of polysorbate may lead to higher levels of high molecular weight (HMW) species, and Fc-region oxidized variants due to autoxidation reactions induced by light exposure. Impurities in polysorbate can act as photosensitizers, absorbing visible light and then contributing to protein degradation.^18^ Moreover, oxidation, induced by factors such as UV light exposure or metal contamination, has also been linked to the formation of aggregates.^22^

The impact of visible light on mAB photodegradation has been reported in the context of exposure within glass bioreactors using 400–800 nm light, which led to methionine oxidation and the increased formation of acidic variants from deamidation.^23^ Blue LED light (400–500 nm) was recently found to be primarily responsible for the ROS formation and aggregation of IgG4 monoclonal antibodies (mAb) in Histidine buffer. Degradation of the histidine buffer under blue light also corresponded to the increased presence of reactive oxygen species and the potential to generate further photosensitizers. They also noted that the photodegradation products of tryptophan residues, such as kynurenine and N-formyl kynurenine, could act as photosensitizers to visible light, and so exacerbate protein damage.^24^ Interestingly, a study of eight mAbs exposed to a range of nominally “visible” light sources, such as cool white fluorescent (CWF) light, revealed that it was actually the low levels of UVA light leakage in these light sources that was the primary reason for aggregation^25^ and that the impact on aggregation could be detected even at only 3 mW/m^2^ of UVA after 30 days.

The impact of formulation excipients, including the protective effects of methionine and tryptophan, on the photodegradation of an IgG1 mAb has also been evaluated. Light exposure according to Q1B guidelines led to up to 10% mAb aggregation resulting from new (non disulfide) covalent linkages formed between chains, along with destabilization of the CH2 domain, and the appearance of two methionine oxidations in the Fc region (CH2 and CH3 domains) that were both close to each other at the CH2-CH3 interface.^26^ Photosensitization to UVA by Tween80, and the protective effects of methionine have also been analyzed for mAbs in recent NMR studies which revealed reactions triggered by light exposure that continue even in the dark.^27^

Fab fragments comprise just four domains (VH, CH1, VL, and CL) from antibodies, and have so far been developed to treat a broad spectrum of diseases, including rheumatoid arthritis, multiple sclerosis, age-related macular degeneration, retinopathy, and thrombosis.^28^ The 47.4 kDa A33 Fab is an undeveloped immunotherapeutic designed to target the human A33 antigen prevalent in primary and metastatic colorectal cancers, and has a known X-ray crystal structure.^29^ It has been used extensively to examine and model aggregation mechanisms during long-term storage,^30^ characterized by small-angle X-ray scattering,^31^ computationally design mutagenesis,^32^ and molecular dynamics simulations.^33^ These studies have collectively revealed that the aggregation kinetics are mainly controlled by partial unfolding events to a native-like N* state, caused by increased flexibility of the heavy-chain C-terminus (hinge region) and loss of native contacts in the CL domain. These events lead to increased exposure of critical aggregation-prone regions as observed in molecular dynamics simulations^33^ and correlated to aggregation kinetics in PLS-based predictive models.^34^

While the general effects of light exposure, agitation, formulation, and temperature on antibody stability have been well characterized, their interactions and molecular mechanisms for Fab fragments are not well understood. In this study, we examined the stability of the A33 Fab fragment to thermal, agitation, and light-induced stresses, in formulations that included Tween 80, using 10 mg/mL Fab which is typical to therapeutic dosage forms such as in Lucentis (see package insert, Genentech 2006). Studies were carried out in polypropylene microplates, with and without siliconization, to represent the materials used in prefilled syringes. Instability was measured as monomer loss and fragmentation using SEC-HPLC, changes of the hydrodynamic radius using DLS, chemical modifications to the intact monomer as determined by mass spectrometry, and N-terminal sequence analysis. Briefly, we found that light exposures resulted in photoinduced clipping of amino acids, selectively for the heavy chain C-terminus, and that these likely promoted routes to fragmentation by chain dissociation, and also to aggregation of the Fab.

## Materials and Methods

### Materials

Tween 80 (polysorbate 80), Sodium phosphate dibasic and Sodium phosphate monobasic salts, silicone oil, Ammonium acetate, and sodium chloride were bought from Merck, Germany.

### Fab Preparation

Fab was expressed in *E. coli* W3110 in a pilot-scale BIOSTAT Cplus 30 L fermenter (Sartorius, Gottingen, Germany) and purified by Protein G chromatography and gel filtration all as described previously.^35^ Purified protein was exchanged into ultrapure water at 4 °C overnight and concentrated to 36.75 mg/mL using 10 kDa MWCO Dialysis Cassettes (Thermo Scientific, UK). Previous analysis of Fab samples prepared in this way were shown to contain no higher molecular weight species, and only very minor levels (<1%) of host cell protein, nondisulfide linked chains, or single chain fragments.^35,36^

### Fab Formulation Preparation

Fab in water was buffer exchanged to 200 mM phosphate, pH 7.5 or 5.5, either with or without 0.26% (w/v) Tween 80, using 30 kDa MWCO filters followed by 0.22 μm filtration (GE Healthcare, USA) to ensure particle free samples. Concentrations were determined by UV–vis spectroscopy from absorbance at 280 nm and an extinction coefficient of 66,329 mM^–1^ cm^–1^ as previously,^30^ then adjusted by dilution in the same buffer to 10 mg/mL Fab. Fab samples were stressed and analyzed (as below) alongside an unincubated (unstressed) reference control prepared at 4 °C and analyzed immediately.

### Accelerated Degradation

100 μL of 10 mg/mL Fab formulations were loaded into 48-well polypropylene microplates that were either untreated or siliconised using a standard baking procedure^37^ in which silicone oil filled plates were incubated at 70 °C overnight. Excess silicone oils were washed extensively with ddH_2_O followed by further drying at 70 °C overnight. Plates were loaded into a thermomixer (Eppendorf, Germany) with Peltier-controlled temperature. Black opaque plastic lids were 3-D printed to the same design as those from the Thermomixer but designed to house one LED directly 5 cm above the sample surface in each well, with separate lids created for each LED color of Blue (BL), Green (GL), Red (RL), Amber (AL), White (WL), or UV light. LEDs were spectrally analyzed offline prior to installations. Given the 15–16° LED viewing angles, 13 mm well diameters, after 72 h, one BL LED of 4.1 cd generated 61.87 W·h/m^2^ luminosity, one GL LED of 5 cd generated 75.63 W·h/m^2^ luminosity, one RL LED 5 cd generated 20.34 W·h/m^2^ luminosity, one AL LED of 5 cd generates 57.04 W·h/m^2^ luminosity, and one WL LED of 25 cd generates 109.58 W·h/m^2^ luminosity. UV luminosity was 1285 W·h/m^2^. These can be compared to the ICH Q1B guidelines of 200 W·h/m^2^. Plates were agitated by orbital shaking at either 300 or 600 rpm at the preset temperature and light exposure, for 72 h prior to measurement of Fab monomer loss and particle sizing.

### Monomer Loss Determination by Size-Exclusion Chromatography

Stressed Fab samples were analyzed alongside the unincubated reference control by size exclusion chromatography using a Zorbax GF-250 4.6 × 250 mm column (Agilent Technologies, Palo Alto, CA, USA) on an Agilent 1200 HPLC with column compartment at 20 °C, detection by Absorbance at 280 nm, an isocratic mobile phase of 0.2 M Na-Phosphate, pH 7, a flow rate of 1 mL/min, and 5 μL injection of 10 mg/mL Fab samples.

### Particle Size Analysis by Dynamic Light Scattering (DLS)

Fab samples at 10 mg/mL were analyzed by DLS (Zetasizer Ultra, Malvern, UK) to measure particle size distribution, and report on the associated average hydrodynamic radius (*Z*_av_) and poly dispersity index (PDI).

### Intact Mass Analysis

Fab samples were buffer exchanged into 0.2 M Na-Phosphate, pH 7.5, using Amicon ultra filters (10 kDa MWCO) to give 50 μL of a 3.85 mg/mL solution (69% protein recovery). For subsequent MS analysis under reduced condition, an aliquot of the product was buffer exchanged twice into ammonium bicarbonate (50 mM, pH 8.0) using two Zeba Spin 6 columns, and the final sample was diluted to a 0.3–0.4 mg/mL protein concentration prior to mass spectrometry analysis.

Characterization of intact protein was performed using an Agilent 6530 QTOF LC-MS/MS (Agilent, UK). Ten μL portion of 0.2 μg/μL sample was injected onto an Agilent PLRP-S (150 mm × 2.1 mm, 1000 Å, 8 μm) column. The column was maintained at 30 °C. Mobile phase A (5% MeCN in aqueous 0.1% formic acid) and B (95% MeCN, 5% water, 0.1% formic acid) were used in gradients as follows:- 25% B for 1 min followed by increase to 90% B over 16 min. After 2 min, 90% of B was decreased to 25% over 0.1 min and maintained at 25% B for 1.9 min. The flow rate was 0.3 mL/min. The QTOF mass spectrometer operated using positive electrospray ionization (ESI) scanning at the *m*/*z* range from 100 to 3200 Da and acquiring profile data. The electrospray source operated with a capillary voltage of 4000, fragmentor at 175, skimmer at 65 and octopole RF peak at 750. Nitrogen was used as the nebulizer and desolvation gas at a flow of 5 L/min. The acquisition rate was 1 spectra/s and acquisition time 1000 ms/spectrum. Lockspray was used during the analysis to maintain mass accuracy. The data was processed using MassHunter software (version B.07.00) and deconvoluted using a maximum entropy deconvolution algorithm.

### N-Terminal Sequencing

Fab samples were prepared at 1 mg/mL by dilution in the same buffer and sent by courier on ice packs, for N-terminal sequencing at Alta Bioscience (Redditch, UK), using Edman degradation and HPLC-based sequencing.^38^

## Results and Discussion

### Initial Condition Screen

An initial scoping study examined the impact of pH (5.5 vs 7.5), and agitation (300 vs 600 rpm) on the monomer loss of 10 mg/mL Fab at 45 °C after 72 h, in nonsiliconised polypropylene 48-well microplates. This simply aimed to define basic experimental conditions that produced measurable monomer losses on a practical time scale of 72 h, prior to wider screening with additional stress conditions such as temperature and light exposure. Samples were analyzed by SEC-HPLC to measure the monomer loss relative to control samples stored in the same buffers for 72 h at 4 °C (unagitated and in the dark). DLS was also used to measure changes in average hydrodynamic radius (*Z*_av_) and polydispersity index (PDI). It was found overall that changing agitation from 300 to 600 rpm, or pH from 5.5 to 7, did not impact monomer loss significantly on this time scale, although the samples at pH 5.5 and 300 rpm had slightly lower monomer loss (12.3 ± 1.5%), than the other three conditions (all 17.2% with s.e.m. ranging 1.2 to 3.7%). Based on this preliminary study, it was decided to fix the agitation to 600 rpm and pH 7.5 for maximum monomer loss.

### Full Factorial Screen

A Design of Experiments (DoE) approach was taken to evaluate the impacts and interactions of temperature (4, 10, 21, 30, 45 °C), LED color (blue, green, amber, red, no light), plate siliconization, and the presence or absence of 0.26% (w/v) (2 mM) Tween80, on A33 Fab monomer loss. The 0.26% (w/v) Tween80 is significantly above the critical micelle concentration (CMC) of approximately 0.0016% (w/v), to ensure complete masking of any free silicone oil or siliconised surfaces, and we have previously found even higher concentrations to have no adverse impact on stability.^39^ The full factorial design required 100 runs (Table S1, Supporting Information), repeated in triplicate, for 10 mg/mL Fab in phosphate buffer, pH 7.5, agitated at 600 rpm for 72 h in polypropylene microplates. Samples were again evaluated by SEC-HPLC for monomer loss and by DLS (*Z*_av_ and PDI). The visible light sources selected were engineered into bespoke lids for the plate-shaking thermomixer, placing one LED over each well of the 48-well microplate. Measurements of LED light emission spectra were made in a UV spectrometer (Figure S1, Supporting Information) to confirm their peak intensity maxima at 469 nm (blue), 522 nm (green), 592 nm (amber), and 632 nm (red).

A comparison of the output data from SEC and DLS (Figure 1) showed that initial monomer losses of 10–25%, as measured by SEC, led to the formation of either no aggregates (*Z*_av_ ∼ 8 nm) or only small aggregates (20–150 nm). Higher monomer losses of >25% then often led to larger particle formation.

**Figure 1:**
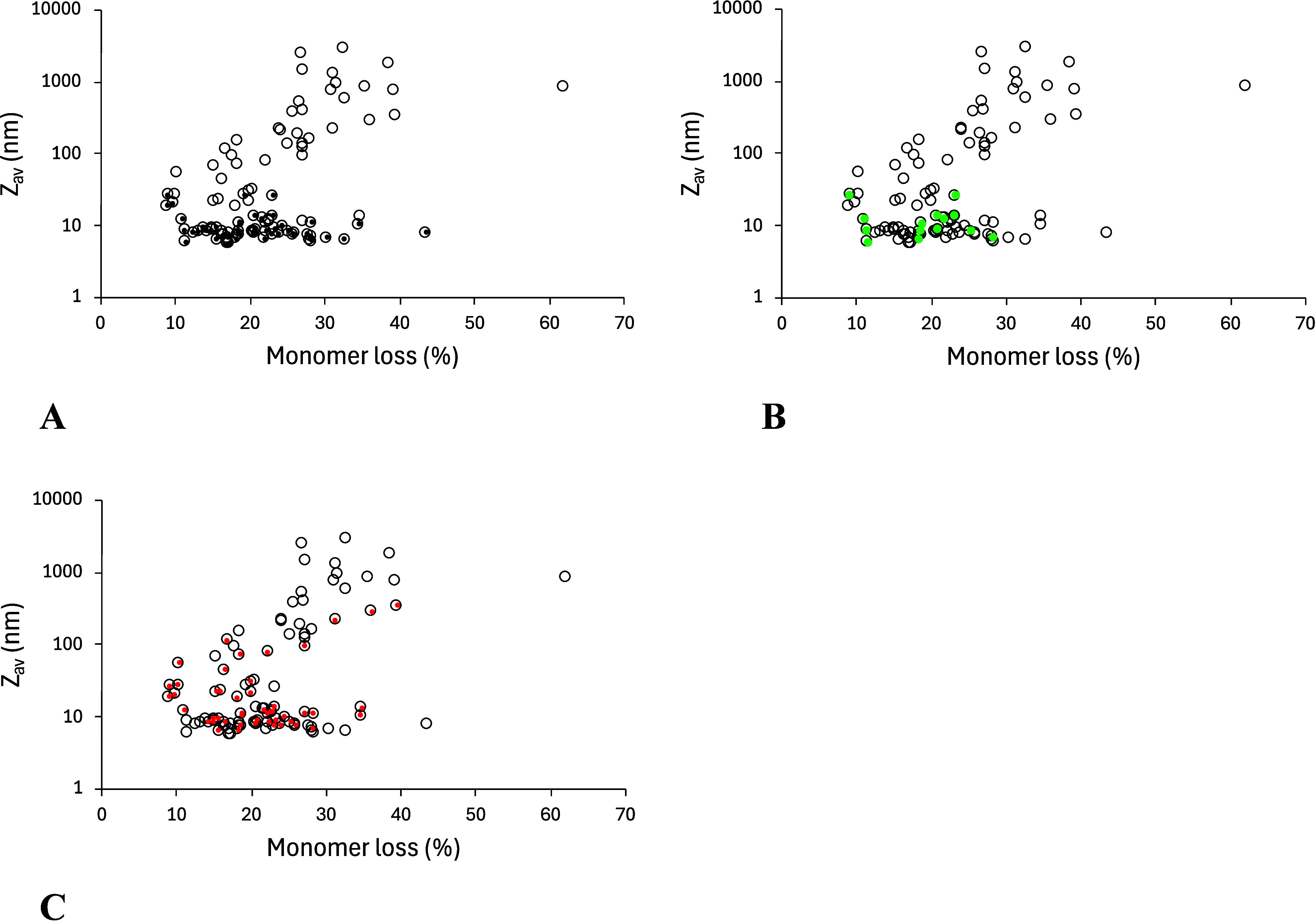
Comparison of monomer losses and average particle sizes (*Z*_av_) as measured by SEC and DLS. 10 mg/mL Fab in phosphate buffer, pH 7.5, was incubated at 600 rpm for 72 h in polypropylene 48-well microplates. Data are from a full factorial screen of temperature (4, 10, 21, 30, 45 °C), LED color (blue, green, amber, red, no light), with/without plate siliconisation, and the presence or absence of 0.26% (w/v) (2 mM) Tween80. (A) Black dots, samples in unsiliconised plates; (B) Green dots, samples that showed fragmentation by SEC; (C) Red dots, samples that contained Tween 80.

Samples that formed large particles observed by DLS were nearly always in siliconised plates. By contrast, the nonsiliconised plates nearly always led to monomer losses with small particles or no significant increase in *Z*_av_ measured by DLS (Figure 1A). In many cases whereby the monomer loss increased but the *Z*_av_ did not change significantly, the SEC-HPLC detected fragmentation of the Fab (Figure 1B and Figure S2, Supporting Information). Samples containing Tween 80 were distributed throughout the cluster of samples having monomer losses but no increase in particle size, so Tween 80 did not appear to suppress fragmentation of the Fab or monomer losses to small aggregates. By comparison, Tween 80 appeared to suppress the largest particle formation observed in samples with siliconization, although not completely.

Analysis in Minitab (Minitab.LLC, USA) of the fitted means from the full DoE data set (Figure 2) showed an overall consistency in terms of the effects of temperature, light sources, Tween 80 and siliconisation, on the monomer loss, *Z*_av_ (nm) and PDI. The main effects were an increased monomer loss and larger particle formation at higher T (see also Figure S3A Supporting Information), in the presence of siliconisation, and absence of Tween 80 as would be expected. The impact of light depended on the specific sources, with blue light being the only one leading to a significant increase in the mean monomer loss (see also Figure S3B Supporting Information).

**Figure 2:**
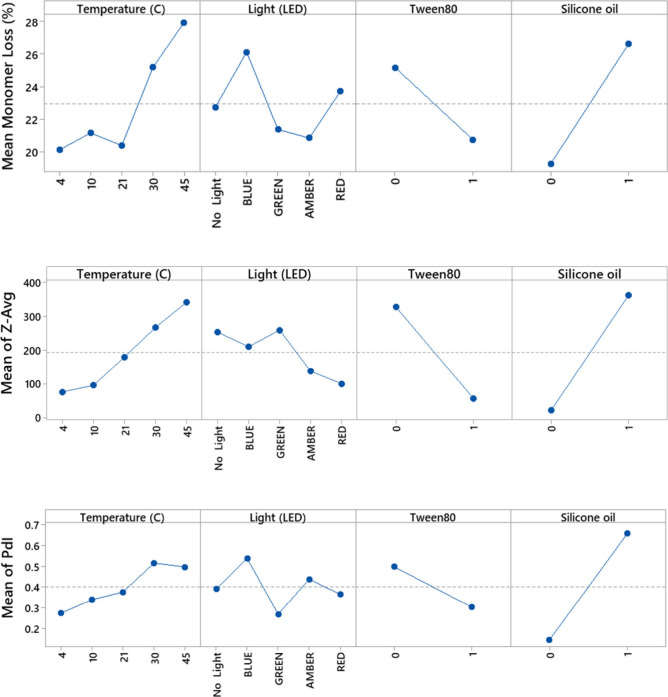
DoE-model fitted means for monomer loss, particle size, and distribution. Fitted mean values are shown as a function of each factor used in the DoE model. Factors were temperature, LED light source, presence/absence (1/0) of Tween 80, or with/without (1/0) silicone oil treated plates. Adjusted *R* ^2^ values for each fitted model were 0.9 (monomer loss), 0.61 (*Z*_av_) and 0.67 (PDI).

Fitted means can be influenced by large changes under only a few specific conditions. Indeed, under most conditions, including “no light”, temperature did not actually have a strong effect on the monomer loss (Figure 3), indicating that the baseline conditions chosen were not harsh enough to elicit large differences on this experimental time scale. This was not unexpected given that the *T*_m_ of A33 Fab is typically >65 °C,^35^ and so global unfolding is still minimal even at 45 °C. Additionally, the kinetics of A33 Fab monomer loss measured previously at 1 mg/mL, pH 7 without agitation were only 0.004% day^–1^ at 4 °C, 0.06% day^–1^ at 45 °C and 2.7% day^–1^ at 65 °C.^30,32^ Thus, the low monomer loss of only 0.18% expected after 72 h at 45 °C, pH 7.5, would still be under the limit of detection for a change in monomer content as measured by SEC. For 10 mg/mL Fab, pH 7.5 and agitation at 600 rpm in microplates, the fitted mean monomer loss and *Z*_av_ overall increased at 30 and 45 °C, (Figure 2) but this was primarily driven by exceptional changes under amber and blue light (at 45 °C) and red light (at both 30 and 45 °C), especially in the presence of silicone oil.

**Figure 3:**
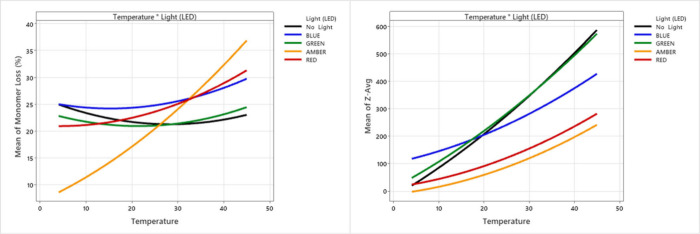
Dependence of DoE-model fitted means on temperature and light source Adjusted *R* ^2^ values for each fitted model were 0.9 (monomer loss) and 0.61 (*Z*_av_).

Overall, light exposures had a relatively minor influence on particle size compared to temperature, Tween 80 and silicone oil. However, the interactions between temperature and light source (Figure 3) showed that blue, amber, and red light led to smaller average particle size (*Z*_av_) at higher temperatures, when compared to no light or green light conditions, despite the increased monomer loss. This indicated that these light sources led to increased fragmentation at higher temperatures, which is also consistent with the observation of fragment peaks by SEC (Figure S2, Supporting Information) more often in samples exposed to red, amber or blue LEDs. The interaction plots based on fitted means in Figure 3 also potentially suggested that the amber light exposure was stabilizing against monomer losses at low temperatures, compared to its destabilizing impact at high temperature. However, the standard deviations across the replicas under each of the low temperature conditions were also high, and so this was likely not a significant effect.

Aside from the impact of light, there were also significant interactions between Tween and siliconisation, temperature and siliconisation, as well as temperature and Tween 80, as might be expected. Siliconisation and higher temperatures each led to greater monomer losses, and larger particle formation, while both effects were counteracted by the addition of Tween 80 (Figure 4). Surface response plots showed that blue light broadly gave the same response as no light in these factors but with greater overall monomer losses under blue light exposure. These plots additionally revealed a region of higher monomer loss at low temperature with siliconisation or absence of Tween 80, in which the *Z*_av_ remained relatively low. Despite the siliconisation, these were additional conditions in which monomer losses did not lead to large particle sizes but instead resulted mainly from increased fragmentation of the Fab with the appearance of up to two new fragment peaks in SEC.

**Figure 4:**
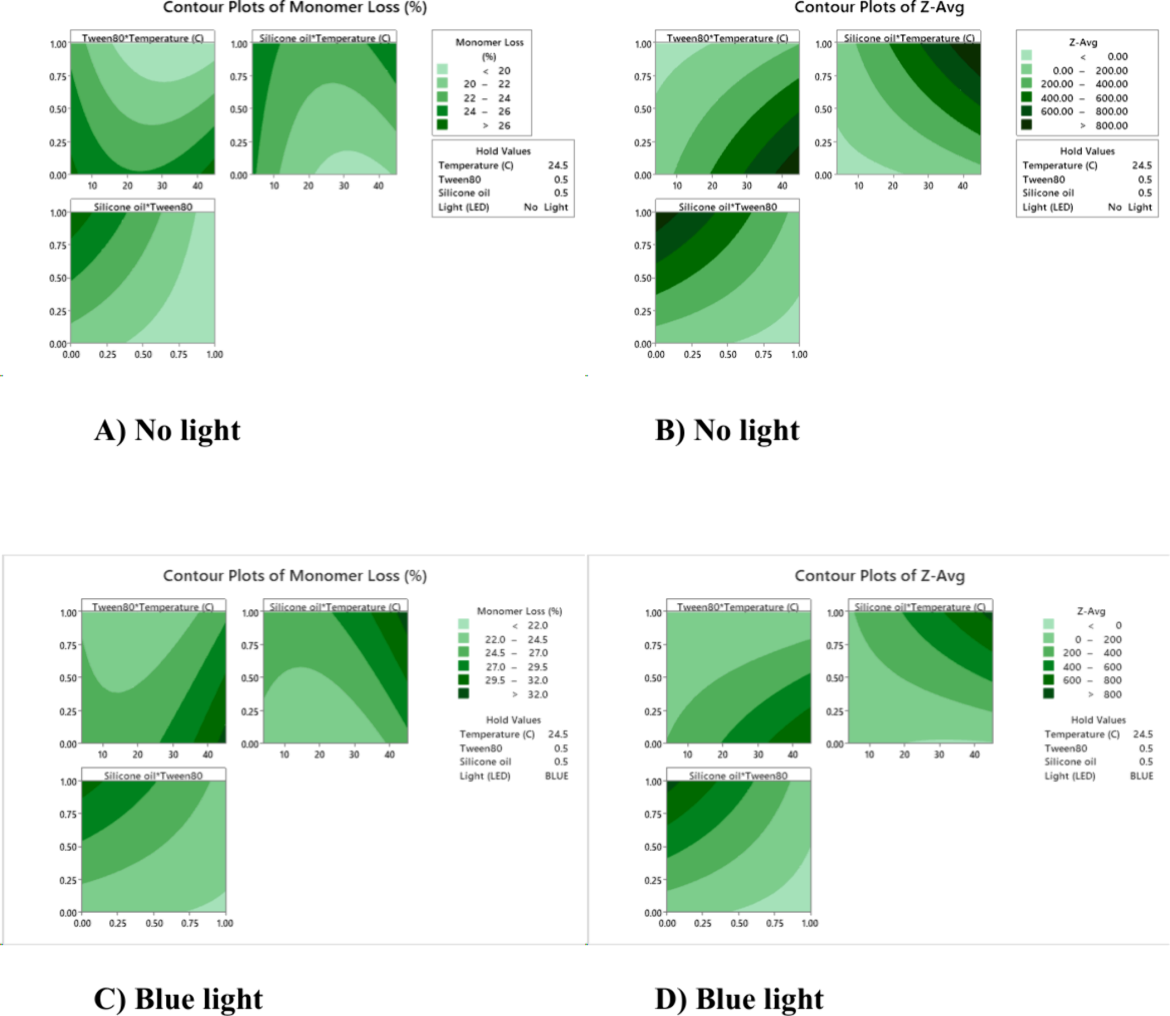
Response surface plots based on the DoE fitted model comparing no light (A and B) and blue light (C and D) exposure. Response surfaces are shown for monomer loss (A and C) and *Z*_av_ (B and D) to show the pairwise interactions between tween 80, temperature and siliconisation, with the third factor held at midpoint values.

### Introducing White and UV Light Exposure

A second full factorial study explored UV (380 nm) and white (400–700 nm range) light sources as these are more closely aligned to those required in ICH guideline Q1B. These were evaluated for monomer losses across the five temperatures, with and without Tween 80, giving a total of 20 new conditions. Siliconisation was dropped to simplify the study and remove its specific influence on large particle formation.

The “dark control” samples that were incubated but with no light exposure were repeated at the same time, along with the unincubated reference control. This ensured that all data could be directly compared to those obtained using the previous light sources under the same experimental settings in nonsiliconised microplates agitated at 600 rpm for 72 h (Figure 5). The highest monomer losses were induced at 45 °C as expected, but this only occurred under exposure to white, UV, amber, and to some degree blue light. Under amber light the monomer losses had high error bars due to significant losses (>50%) occurring in only one or two of the replicas, indicating some stochasticity in the emergence of aggregates. While the increased monomer loss at high temperature remained significant despite this variability, the apparently decreased monomer loss at low temperatures under amber light still fell within the error margins.

**Figure 5:**
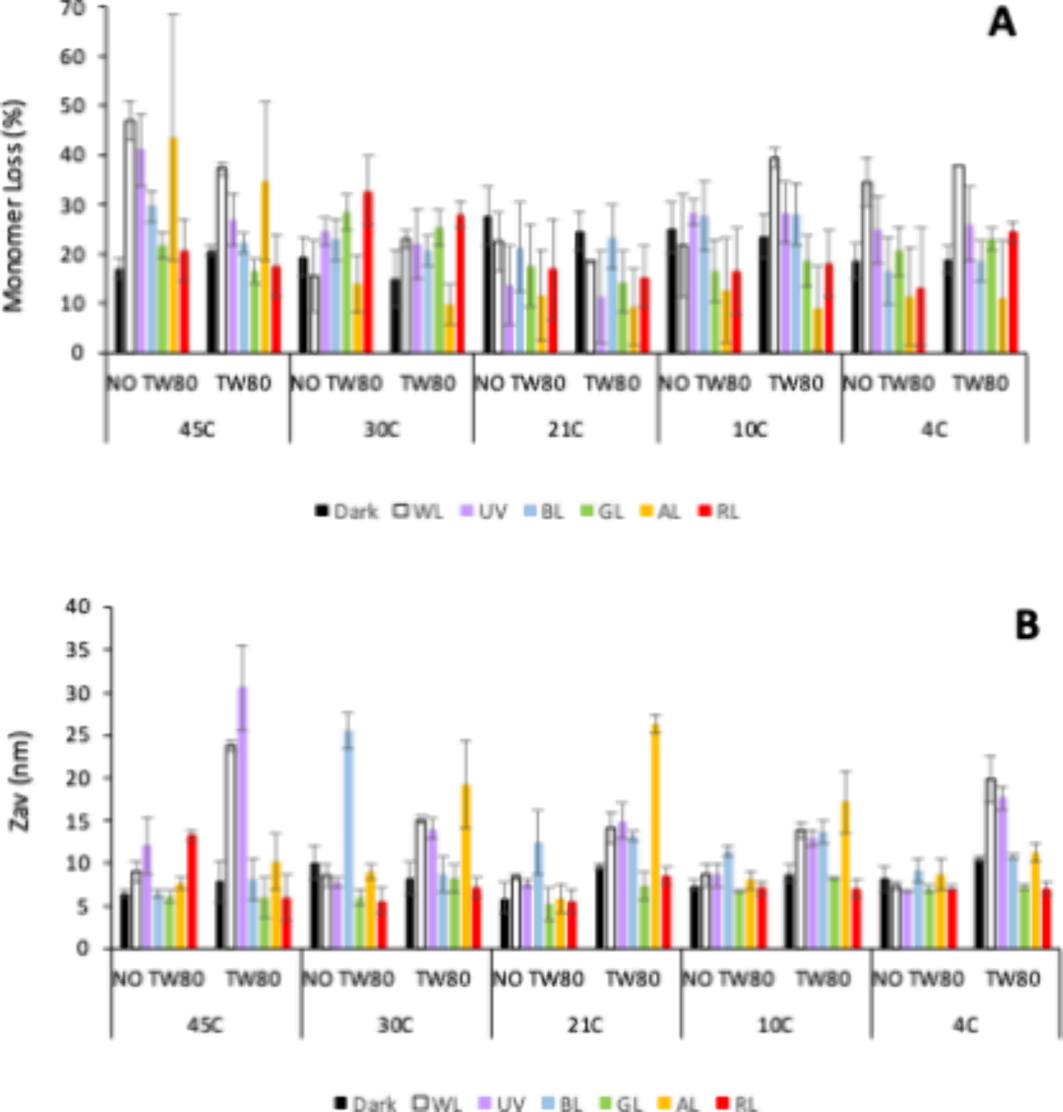
Comparison of monomer losses for all light sources as measured by (A) SEC and (B) DLS *Z*_av_. Samples were analyzed by SEC and DLS after 72 h in unsiliconised 48-well polypropylene microplates, with agitation at 600 rpm, for 10 mg/mL Fab in phosphate buffer, pH 7.5, in the presence (TW80) or absence (NO TW80) of 0.26% (w/v) (2 mM) Tween 80.

Exposure to red and green light under the nonsiliconised conditions only impacted monomer loss at 30 °C, but the differences were small and remained close to the margins of error when comparing to monomer losses obtain with no light exposure. Unusually, for the white light and possibly UV exposure conditions, the monomer loss also increased at 4 and 10 °C. The presence of Tween 80 had little impact on this monomer loss and even potentially increased it in some cases, suggesting that aggregation or plate surface interactions were not primary mechanisms. Indeed, SEC chromatograms showed that white light and UV exposed samples at 45 and 4 °C in particular had increased fragmentation. For comparison, Tween 80 reduced monomer losses in all cases at 45 °C, including those where it was exacerbated by light exposure. This is the expected impact of such a detergent on either aggregation or losses that occur through surface interactions.

Now in the absence of siliconization, the formation of aggregates was much lower across all conditions, such that *Z*_av_ remained essentially constant in the range 6.5–10 nm. In the presence of Tween 80, particle sizes were slightly higher for white light and UV-exposed samples in particular. However, they only reached *Z*_av_ values of 15–30 nm, compared to values of >200 nm in the previous DoE experiments containing siliconised plates, indicating that aggregation, though present, was not the main cause of monomer loss. Instead, as confirmed by SEC, fragmentation was a significant cause of monomer losses. However, the presence of Tween 80 appeared to direct the impact of UV and white light sources toward some aggregate formation in addition as measured by DLS.

Overall, the main source of increased monomer loss at high temperature was exposure to white, UV, or blue LEDs, with the strength of the effect in that order and fragmentation prominent with these light sources. This indicates that shorter wavelengths in the blue to UV range are likely to have the main impact. It should be noted that the white light source was measured to have high intensity peaks at 452 nm (blue) and a broader peak centered at 535 nm but spanning 500–650 nm (green to amber), but no peak equivalent to the UV LED (369 nm). However, the white light LED intensity at 369 nm was not zero and remained at 300-fold lower than for the UV LED. Furthermore, the UV, blue, and white light LEDs all had nonzero background intensities at 200–340 nm, which is the critical range for absorbance by protein amino acids, and so potentially, it is exposure to these wavelengths with all three of these LEDs that led to photodegradation and subsequent monomer losses. This observation would be consistent with previous studies of mAbs exposed to different “white” light sources, which revealed UV-A (315–400 nm) light leakage in these sources was responsible for aggregation, which could be detected even at only 3 mW/m^2^ of UV-A after 30 days.^25^ The photodegradation effect of UV-A is well documented, with UV-A light exposure for 72 h leading to 4–12% mAb monomer loss,^40^ and in other studies with UV-A light exposure of mAbs leading to 12–13% of aggregates and fragments formation.^25,41^

In our work, based on the areas under the spectra in Figure S1 (supplementary), the light exposures over 72 h from only the 200–340 nm range, would be equivalent to 0.07 W·h/m^2^ (Blue), 0.1 W·h/m^2^ (White), and 9 W·h/m^2^ (UV), and so relatively low compared to ICH Q1B guidelines of 200 W·h/m^2^ for UV-A, but closer to the exposures of 2 W·h/m^2^ previously reported to aggregate mAbs.^25^

### Intact Mass Analysis of Fab

To get a more detailed structural analysis of monomer loss due to external stress exposure, intact masses of Fab samples were analyzed by mass spectrometry using a QTOF LC-MS with electrospray ionization after peak separation of protein species by reversed-phase HPLC. Samples were incubated as above at 21 °C for 72 h with different light exposures in the presence or absence of Tween 80. This temperature was chosen because it led to the lowest monomer losses through aggregation and so aimed to minimize the formation of larger particles by any light-damaged species that would then not enter the mass analysis.

From the primary HPLC chromatogram, it was evident that stress-exposed samples had more than one peak compared to the control Fab, with the main peak eluted at 3.1 min, the appearance of a tailing shoulder on the main peak at 3.25 min, and two overlapping small peaks at 2.7–2.8 min. The Fab control had a single group of intact masses with a major mass peak of 47388 Da after deconvolution from the HPLC peak eluted at 3.1 min (Table 1 and Figure S4, Supporting Information). There were also several smaller peaks of higher mass, each separated by 16 Da indicating a degree of heterogeneity in the control sample intact mass, most likely from oxidations during production and storage. For stressed samples, no changes were observed in this overall intact mass distribution when compared to the Fab control and so stress with light exposures at 21 °C for 72 h did not significantly increase the oxidation state of the Fab. However, mass-deconvolution of the primary HPLC peak (3.1 min) did reveal some significant mass losses within the intact Fab monomer that had occurred due to chemical modifications resulting from the external light source exposure.

**Table 1:** Intact Mass Analysis of A33 Fab after Light Exposures^a^

Sample	Detected Mass (Da)	Total mass loss (Da)	Step mass loss (Da)
Control	47387.76	0	
BL	47244.68	143.1	143.1
	47056.38	331.4	188.3
	46818.06	569.7	238.3
BL, TW80	47244.45	143.3	143.3
	47056.36	331.4	188.1
	46818.05	569.7	238.3
Dark, TW80	47387.12	0.6	0.6
	47245.52	142.2	141.6
GL	47244.5	143.2	143.3
	47056.62	331.1	187.9
	46818.04	569.7	238.6
GL, TW80	47386.58	1.2	1.2
	47244.06	143.7	142.5
	47056.62	331.1	187.4
	46818.12	569.6	238.5
WL	47387.22	0.5	0.5
	47244.86	142.9	142.4
WL, TW80	47387.22	0.5	0.5
	47244.86	142.9	142.4
	47056.33	331.4	188.5
UV	47387.22	0.5	0.5
	47244.86	142.9	142.4

a 10 mg/mL Fab in phosphate buffer, pH 7.5, was incubated at 600 rpm for 72 h in unsiliconised polypropylene 48-well microplates at 21 °C, under no light, or blue (BL), green (GL), UV or white (WL) LEDs, and in the presence (TW80) or absence of 0.26% (w/v) (2 mM) Tween80. The Fab control sample was not agitated and was stored in the dark at 4 °C.

After incubation at 21 °C, for 72 h in the presence of Tween 80, the main Fab HPLC peak deconvoluted into two groups of masses, with the first the same as the control (47388 Da) but the second at 47245 Da, indicating a loss of 143 Da. The light-stressed Fab samples all retained the 47245 Da mass, while blue light (with and without Tween 80) and green light (without Tween 80) each led to the loss of the original 47388 Da mass group. Given that the relative peak sizes of the 47388 and 47245 Da masses appeared to vary, even for the Fab incubated without light exposure, this indicated a mass shift resulting from the removal of one or more adducts, or a chemical modification during the incubations at 21 °C.

Light exposures with blue light and to lessening degrees with green and white light led to the emergence of a lower mass of 47057 Da, while blue light in particular also led to a mass of 46818 Da, especially in the presence of Tween 80. We cannot rule out that these lower mass species were not generated by exposure also by white light and UV light. The monomer loss kinetics indicate that these light sources are more problematic, so it remains possible that the lower mass species generated under those light sources were already removed from the mass analysis through aggregate formation. For these reasons, the peak areas from LC-MS cannot be considered to be quantitative, and we can use only the appearance of peaks to identify possible species resulting from light damage. The increased light sensitivity in the presence of Tween 80 has been observed by others previously with monoclonal antibody formulations, due to the formation of peroxide via photodegradation of Tween 80.^19,42^ Hence Tween 80 can act as a photosensitizer. These observations are consistent with the slightly increased *Z*_av_ values for samples containing Tween 80 and exposed to white, UV and to some extent blue light sources, but we cannot establish a clear cause and effect between the mass losses and the formation of the aggregates or fragments observed by SEC and DLS.

The four identified near-native masses corresponded to sequential step mass losses of 143, 188, and 239 Da (Table 1). These were of a magnitude that suggested amino acid clipping from an N- or C-terminus. The heavy and light chain N-termini have sequences of EVQLVES and DIQMTQ, respectively, while the heavy and light chain C-termini have final sequences of CDKTHTSAA and SFNRGEC, respectively. Analysis of these shows that only the heavy chain C-terminus (CDK­THT­SAA) can explain the observed mass losses. Removal of the first two amino acids, AA, corresponded to a mass loss of 142.2 Da. The next two amino acids, TS, corresponded to a further mass loss of 188.1 Da, and the following two, TH, corresponded to an additional loss of 238.2 Da. Thus, the exposure of the Fab to light led to specific peptide bind cleavage events. Such events could also be the result of trace metal-catalyzed reactions, though the impact of light exposure would suggest that any role of trace metal ions if present, would be linked to a photoactivated mechanism. The removal of sequential pairs of residues suggests that the photoinduced lysis of the peptide bonds is either directional in the structure relative to a photoactivatable center, or that it results from an underlying patterning of the structure such as would vary the solvent exposure of the respective peptide bonds. This would be possible with the partially helical nature of the heavy chain C-terminus (Figure 6). It was intriguing that only the heavy chain C-terminus was affected, but not the two N-termini or the light-chain C-terminus. Amino acids that are known to be particularly photosensitive include tryptophan, tyrosine, phenylalanine and cysteine/cystine.^43^ Type I photodegradation begins with absorption of light by these side chains or the peptide backbone, resulting in excitation of an electron to higher energy singlet states. In aqueous solution the absorption wavelengths for these are 180–230 nm for the peptide backbone, 280–305 nm for Trp, 260–290 nm for Tyr, 240–270 nm for Phe, and 250–300 nm for cystine. Increased solvent exposure can promote susceptibility to photodegradation, especially when light absorption involves excipients, buffer or trace metal ions that mediate the formation of reactive oxygen species.^44^ The heavy chain C-terminus of A33 Fab is highly solvent exposed and packs onto the light chain structure to partially bury and stabilize the light-chain C-terminus. The C-terminal region of Fab in the light chain (LC) also contains a cluster of one tryptophan (LC: W148), two tyrosines (LC: Y186, Y192) and three phenylalanines (LC: F116, F118, F209) that could potentially act as a photoactivatable site in the UV range (Figure 6), leading to formation of a localized solvated electron that can migrate to, react with, and lyse nearby peptide bonds or disulfides.^43^ Disulfides are also inherently photosensitive.^45^ The heavy and light chain C-termini are bridged by a disulfide bond between the C-terminal light chain cysteine (SFN­RGE**C**), and cysteine 220 in the heavy chain C-terminus (**C**DK­THT­SAA). Photolysis of this disulfide bond would leave the heavy chain terminus in a more solvent exposed and highly flexible state, leaving it more prone to photolytic cleavage of the peptide bond mediated by buffer components. Meanwhile, dissociation of A33 Fab into the heavy and light chain fragments, as observed by SEC and the LC-MS, could have resulted from either peptide bond cleavages at sites N-terminal to the cysteine residues, or from disulfide bond photolysis.

**Figure 6:**
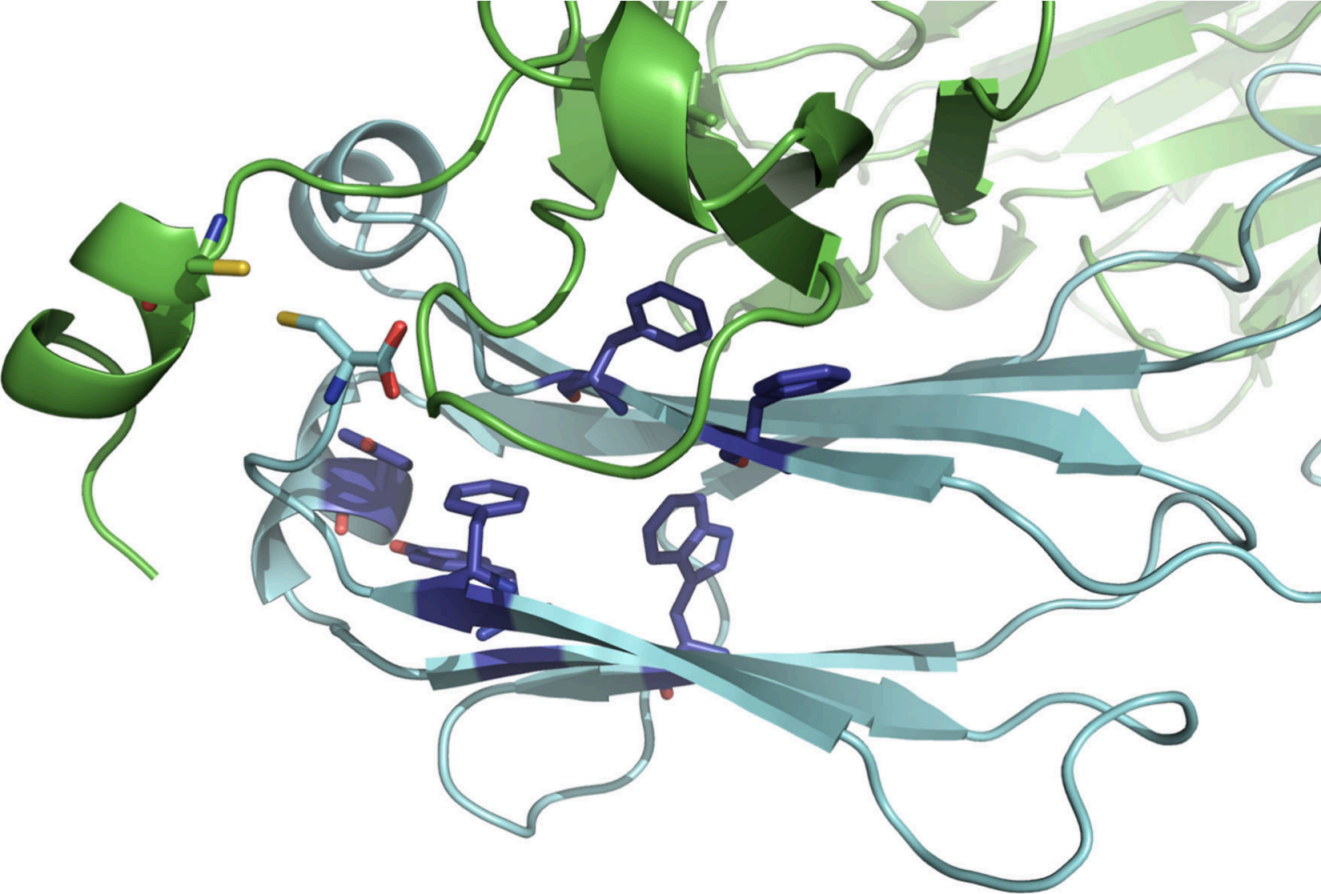
C-terminal region of the Fab structure showing a cluster of photosensitive residues close to the hinge region disulfide. Heavy chain is shown in green, and light chain is shown in blue. Light chain residues F116, F118, W148, Y186, Y192, and F209 are shown as darker blue sticks. The hinge disulfide is shown in the reduced state with the two cysteine residues shown as sticks and sulfur atoms in yellow.

Stressed samples had two additional overlapping peaks in the HPLC at 2.7–2.8 min (from LC-MS), as well as a tailing shoulder in the main peak (3.25 min) that were not present for the Fab dark control (Figure S5, Supporting Information. The shoulder HPLC peak revealed a major mass of 94490 Da which corresponded to a dimer of the first monomer degradation product at 47244 Da (heavy chain AA clipped). The shoulder also contained a minor population of 92953 Da which corresponded to a dimer in which the heavy chains were clipped to remove the peptide CDK­THT­SAA. This clipping was not observed directly in the main monomer peak, indicating that it occurred only in species that had already dimerized, possibly with an interheavy chain disulfide via the clipped cysteine. Rapid dimerization of the monomer after clipping of CDK­THT­SAA appears to be less likely as there were no dimer masses observed that corresponded to Fab monomers with two different heavy chain lengths.

The smaller unresolved HPLC peaks at 2.7–2.8 min revealed a mass peak of 23568 Da, which corresponded to the intact light chain with four oxidations (23565 Da). This observation suggested that fragmentation was induced by photo-oxidation of multiple residues, presumably including the disulfide bond between the heavy and light chain. The masses of the expected unmodified heavy chain and light chain were not observed. Neither did we find any large subfragments of significantly less than 23 kDa that would have suggested peptide clipping from deeper within the protein chain sequences that could also lead to fragmentation. Photolysis of the hinge region disulfide bond, or its removal via further heavy chain C-terminal clipping, would generally promote the separation of A33 Fab into the heavy and light chain fragments as described above. Therefore, it seems likely that the C-terminal clipping observed by LC-MS, is closely linked to the fragmentation observed by both LCMS and SEC, for samples exposed to UV-A containing light sources.

Interestingly, we have previously demonstrated that the aggregation kinetics of A33 Fab are also particularly sensitive to the dynamics of the heavy chain C-terminal region,^35,32^ and so it is very possible that photoinduced C-terminal clipping and/or disulfide photolysis also promotes aggregation. Furthermore, sequential mutagenesis of the C-terminus to remove each of the first 8 residues has also shown that aggregation kinetics increase to a maximum for variant “TGA225” which removed TSAA and “TGA224” which removed HTSAA.^36^

### N-Terminal Sequencing Analysis

As an alternative approach to assess the level of photodegradation of Fab, some of the photostressed samples were analyzed using N-terminal sequencing of the first seven amino acids in the heavy and light chains combined. The most affected samples were chosen based on their high monomer losses.

It was found that compared to the Fab control, N terminal sequence [H-chain: Asp-Ile-Gln-Met-Thr-Gln-Ser; L-chain: Glu-Val-Gln-Leu-Val-Glu-Ser], no significant differences were found in any of the stressed Fab samples, which included exposures to light sources in the presence of Tween 80. This also confirmed the absence of any mass changes observed by LC-MS resulting from amino acid clipping of the N-termini. However, it should be noted that while there were variations in the relative peak intensities of the amino acids at each cycle of Edman degradation, much of the variability is inherent to the method, which is, therefore, not very sensitive to partial changes in which a small population of an amino acid is chemically modified.

It is not yet known whether the observations above can be generalized to other Fab sequences, nor whether the non-natural C-terminus of amino acids SAA are more susceptible to photoinduced clipping. However, for A33 Fab formulated at pH 7.5 we have found that biophysical stability against truncation, aggregation, and fragmentation is best preserved in the absence of Tween 80, avoidance of silicone oils, and using storage in the dark at 4 °C.

The initial conditions of pH 7.5 and 600 rpm were selected to induce more aggregation over 72 h, but aggregation is minimized at pH 5.5.^30^ Siliconisation should be avoided if possible, as it led to the formation of large particles as observed by DLS. If not possible, then the addition of Tween 80 would at least reduce the size of the particles formed. Tween 80 only marginally suppressed monomer losses to either fragmentation or small aggregate formation. Storage in the dark or possibly under amber light was ideal with aggregation promoted by light sources. The presence of Tween80 also further enhanced the formation of C-terminal truncates upon light exposure, particularly for blue light. In summary, the final formulation of A33 Fab would be at pH 5.5, avoid agitation and siliconization, remain in the dark (or amber container), avoid Tween 80 and be kept at 4 °C. In practice, storage in siliconized prefilled syringes, and transportation that cannot ensure zero agitation, would require the addition of sufficient Tween80 to protect against large particle formation, while retaining storage at 4 °C in the dark to avoid its impact on photosensitization.

## Conclusion

This study provides critical insights into how environmental factors, such as light, temperature, and agitation, contribute to and combine to the structural fragmentation and aggregation of a Fab antibody fragment. Our findings demonstrate that light exposure, most likely in the UV-A range, significantly impacts protein stability, with observable C-terminal truncations, chain dissociation, and aggregation contributing to overall monomer loss. The C-terminal truncations would increase the solvent exposure of the hinge-region disulfide bond between the heavy and light chains and so promote reduction and chain dissociation. Alternatively, photoactivation within the local structural region could potentially also photolyze the disulfide bond, though we did not observe this directly.

Light exposure resulted in photoinduced clipping of amino acids that was selective for only the heavy chain C-terminus, and structurally directed to clip at every two amino acids. This strongly suggested that a cluster of nearby aromatic amino acids in the Fab structure was responsible for the local light absorption and photolysis of nearby peptide bonds.

While Tween 80 offered some protection, its stabilizing effect was limited mainly to suppression of large particle formation within siliconised plates. However, there was some evidence that Tween 80 actually promoted photodegradation in samples exposed to white, blue, and UV LED light.

These results demonstrate the complex interplay between photoinduced degradations, fragmentation, and aggregation in antibody fragments and further emphasize the need for stringent control of external stresses in their handling and storage to ensure their efficacy and safety.

## Supplementary Material



## Notes

UV–vis spectral emissions for all LEDs used; table of conditions used and results for design of experiments; size exclusion chromatograms from SEC-HPLC after 72 h incubations of 10 mg/mL Fab in various conditions; monomer losses and particle sizes plotted by light source and by temperature; deconvoluted intact mass analysis of Fab; reverse-phase chromatograms from LC-MS analysis (PDF)
