# Supplementary material for: Stability Testing Reveals Photoselective Clipping of Heavy Chain C‑Terminal Amino Acids That Leads to Fragmentation and Aggregation of an Antibody Fab Fragment
Source: Mol Pharm. 2025 Sep 10;22(10):5939–51. doi: 10.1021/acs.molpharmaceut.5c00592 (PMC12505263; doi:10.1021/acs.molpharmaceut.5c00592)
Supplement: Supplementary file 1 [file mp5c00592_si_001.pdf]

Supplementary information for

Stability testing reveals photo-selective clipping of heavy chain C-terminal amino acids that leads to fragmentation and aggregation of an antibody Fab fragment

Arka Mukhopadhyay[1]; Kersti Karu[2]; Paul A. Dalby[1]*

[1]Department of Biochemical Engineering, University College London, Gower Street, London, WC1E 6BT, UK

[2]Department of Chemistry, University College London, 20 Gordon Street, London, WC1H 0AJ

**Corresponding author*

Figure S1. UV-Vis spectral emissions for all LEDs used. Each has a different output power and so spectra have been normalised to their maximum emissions for comparison purposes.

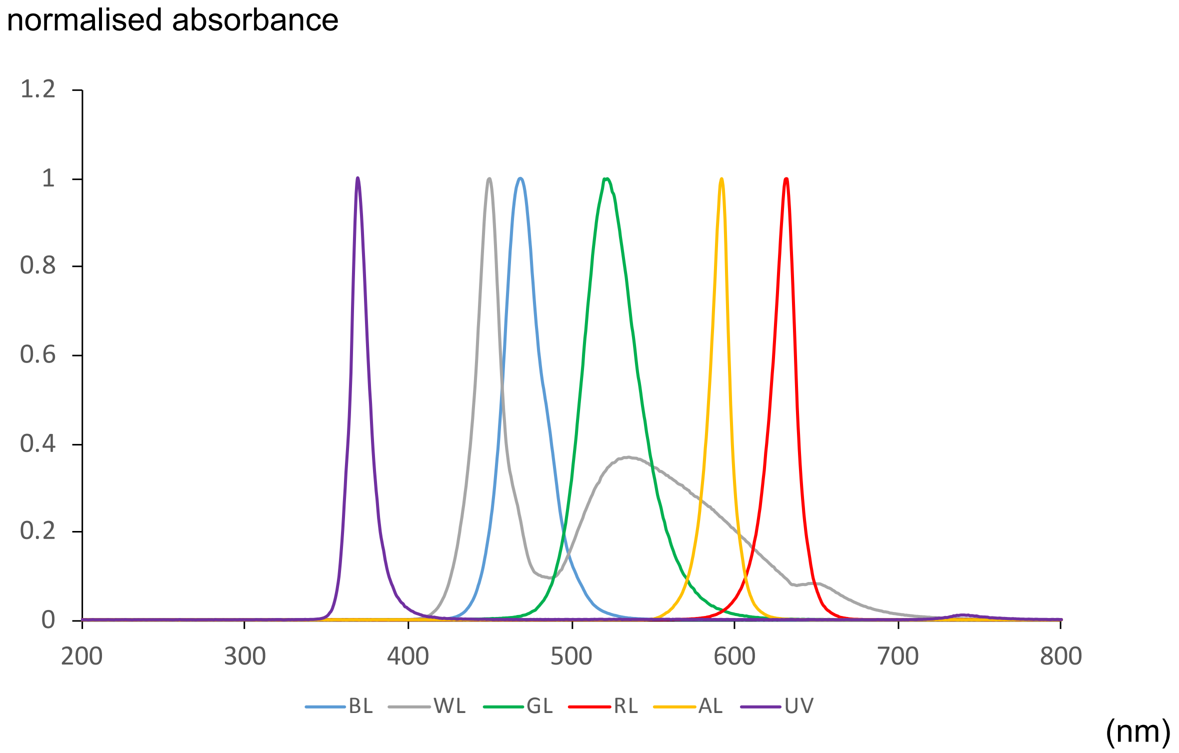

Table S1. Conditions used and results for Design of Experiments: 4 factor, 5,5,2,2 level, full factorial design of experiments. Measured monomer loss, PDI, and Z_{av}.

Run	T (C)	Light (LED)	Tween 80	Silicone oil	Monomer Loss (%)	Z_{av}	PdI
1	4	No Light	0	0	18.6	8.2	0.12
2	4	No Light	0	1	28.2	160.7	0.31
3	4	No Light	1	0	18.8	10.5	0.31
4	4	No Light	1	1	18.2	17.7	0.40
5	4	BLUE	0	0	17.3	7.6	0.13
6	4	BLUE	0	1	25.8	379.4	1.00
7	4	BLUE	1	0	18.8	10.8	0.27
8	4	BLUE	1	1	22.9	11.7	0.44
9	4	GREEN	0	0	20.7	7.7	0.04
10	4	GREEN	0	1	27.3	134.4	0.27
11	4	GREEN	1	0	23.1	7.4	0.13
12	4	GREEN	1	1	22.8	11.2	0.33
13	4	AMBER	0	0	11.5	8.5	0.26
14	4	AMBER	0	1	20.5	31.0	1.00
15	4	AMBER	1	0	11.1	11.9	0.44
16	4	AMBER	1	1	16.5	43.1	0.36
17	4	RED	0	0	13.3	8.0	0.21
18	4	RED	0	1	27.3	120.0	0.33
19	4	RED	1	0	24.0	7.5	0.13
20	4	RED	1	1	20.1	21.2	0.72
21	10	No Light	0	0	26.0	7.4	0.21
22	10	No Light	0	1	27.2	392.8	1.00
23	10	No Light	1	0	23.4	8.8	0.27
24	10	No Light	1	1	21.8	12.3	0.44
25	10	BLUE	0	0	27.8	7.5	0.13
26	10	BLUE	0	1	32.8	555.1	0.98
27	10	BLUE	1	0	28.4	10.7	0.34
28	10	BLUE	1	1	27.3	10.9	0.36
29	10	GREEN	0	0	16.6	7.1	0.05
30	10	GREEN	0	1	26.6	186.9	0.41
31	10	GREEN	1	0	18.7	7.5	0.20
32	10	GREEN	1	1	16.5	8.2	0.27
33	10	AMBER	0	0	12.7	7.8	0.24
34	10	AMBER	0	1	15.4	65.9	1.00
35	10	AMBER	1	0	9.1	18.3	0.50
36	10	AMBER	1	1	10.3	26.5	0.58
37	10	RED	0	0	16.6	7.1	0.01
38	10	RED	0	1	25.2	131.6	0.62
39	10	RED	1	0	18.6	7.3	0.16
40	10	RED	1	1	20.6	8.1	0.25
41	21	No Light	0	0	28.4	5.9	0.16
42	21	No Light	0	1	31.1	736.8	0.64
43	21	No Light	1	0	24.5	9.6	0.25
44	21	No Light	1	1	20.0	30.0	0.40
45	21	BLUE	0	0	21.8	12.4	0.71
46	21	BLUE	0	1	31.4	1268.7	1.00
47	21	BLUE	1	0	23.3	13.2	0.39
48	21	BLUE	1	1	22.3	10.8	0.48
49	21	GREEN	0	0	17.5	5.5	0.03
50	21	GREEN	0	1	24.2	207.8	0.57
51	21	GREEN	1	0	14.4	8.2	0.21
52	21	GREEN	1	1	15.4	21.4	0.80
53	21	AMBER	0	0	11.6	5.9	0.22
54	21	AMBER	0	1	17.9	91.8	1.00
55	21	AMBER	1	0	9.3	26.4	0.26
56	21	AMBER	1	1	10.5	54.4	0.85

57	21	RED	0	0	17.1	5.4	0.04
58	21	RED	0	1	24.2	212.1	0.95
59	21	RED	1	0	15.8	9.0	0.20
60	21	RED	1	1	15.1	9.1	0.40
61	30	No Light	0	0	19.5	26.1	0.74
62	30	No Light	0	1	31.7	935.2	0.60
63	30	No Light	1	0	15.1	8.3	0.21
64	30	No Light	1	1	22.3	76.3	0.67
65	30	BLUE	0	0	23.3	25.5	0.26
66	30	BLUE	0	1	26.9	519.4	1.00
67	30	BLUE	1	0	21.0	8.7	0.24
68	30	BLUE	1	1	26.1	7.6	0.23
69	30	GREEN	0	0	28.2	6.3	0.05
70	30	GREEN	0	1	32.7	2864.0	0.35
71	30	GREEN	1	0	25.4	8.1	0.30
72	30	GREEN	1	1	31.3	211.2	0.70
73	30	AMBER	0	0	13.9	9.1	0.36
74	30	AMBER	0	1	18.6	146.7	1.00
75	30	AMBER	1	0	9.9	19.7	0.49
76	30	AMBER	1	1	16.0	22.4	0.68
77	30	RED	0	0	32.8	6.3	0.05
78	30	RED	0	1	39.3	728.3	0.57
79	30	RED	1	0	28.3	6.9	0.15
80	30	RED	1	1	34.9	12.9	0.43
81	45	No Light	0	0	17.2	6.5	0.08
82	45	No Light	0	1	26.9	2466.7	0.28
83	45	No Light	1	0	20.7	7.9	0.22
84	45	No Light	1	1	16.9	111.8	0.58
85	45	BLUE	0	0	30.5	6.5	0.10
86	45	BLUE	0	1	38.6	1769.9	0.32
87	45	BLUE	1	0	22.4	8.1	0.28
88	45	BLUE	1	1	27.2	92.9	0.33
89	45	GREEN	0	0	22.1	6.4	0.09
90	45	GREEN	0	1	27.3	1450.2	0.51
91	45	GREEN	1	0	15.7	6.3	0.14
92	45	GREEN	1	1	18.6	69.8	0.35
93	45	AMBER	0	0	43.7	7.8	0.26
94	45	AMBER	0	1	62.1	811.3	0.53
95	45	AMBER	1	0	34.7	10.3	0.33
96	45	AMBER	1	1	39.6	340.2	0.48
97	45	RED	0	0	20.8	13.2	0.18
98	45	RED	0	1	35.6	809.3	0.41
99	45	RED	1	0	18.4	6.4	0.20
100	45	RED	1	1	36.1	280.3	0.56

Figure S2. SEC-HPLC after 72 hour incubations of 10 mg/ml Fab, in 200 mM phosphate pH 7.5, with 600 rpm agitation on non-siliconised plates, under no light (dark), white light (WL), UV light (UV), blue light (BL), green light (GL), amber light (AL) or red light (RL), in the absence or presence of Tween 80 (TW), at 4, 10, 21 or 45 °C. The Fab control (C) was stored in 200 mM phosphate pH 7.5 at 4 °C with no agitation or light exposure. Only one of the three replica traces is shown for each sample.

A) SEC of control (no agitation or light, 4C, 200mM Phosphate, pH 7.5)

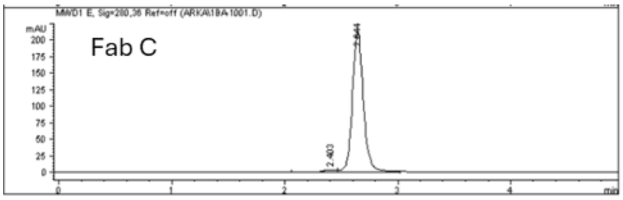

Control for BL, GL, AL, RL study

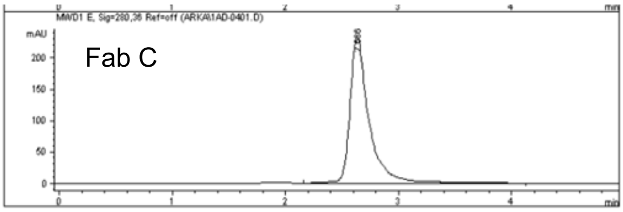

Control for WL, UV study

B) SEC after 45C, 600rpm, 72h, 200mM Phosphate, pH 7.5

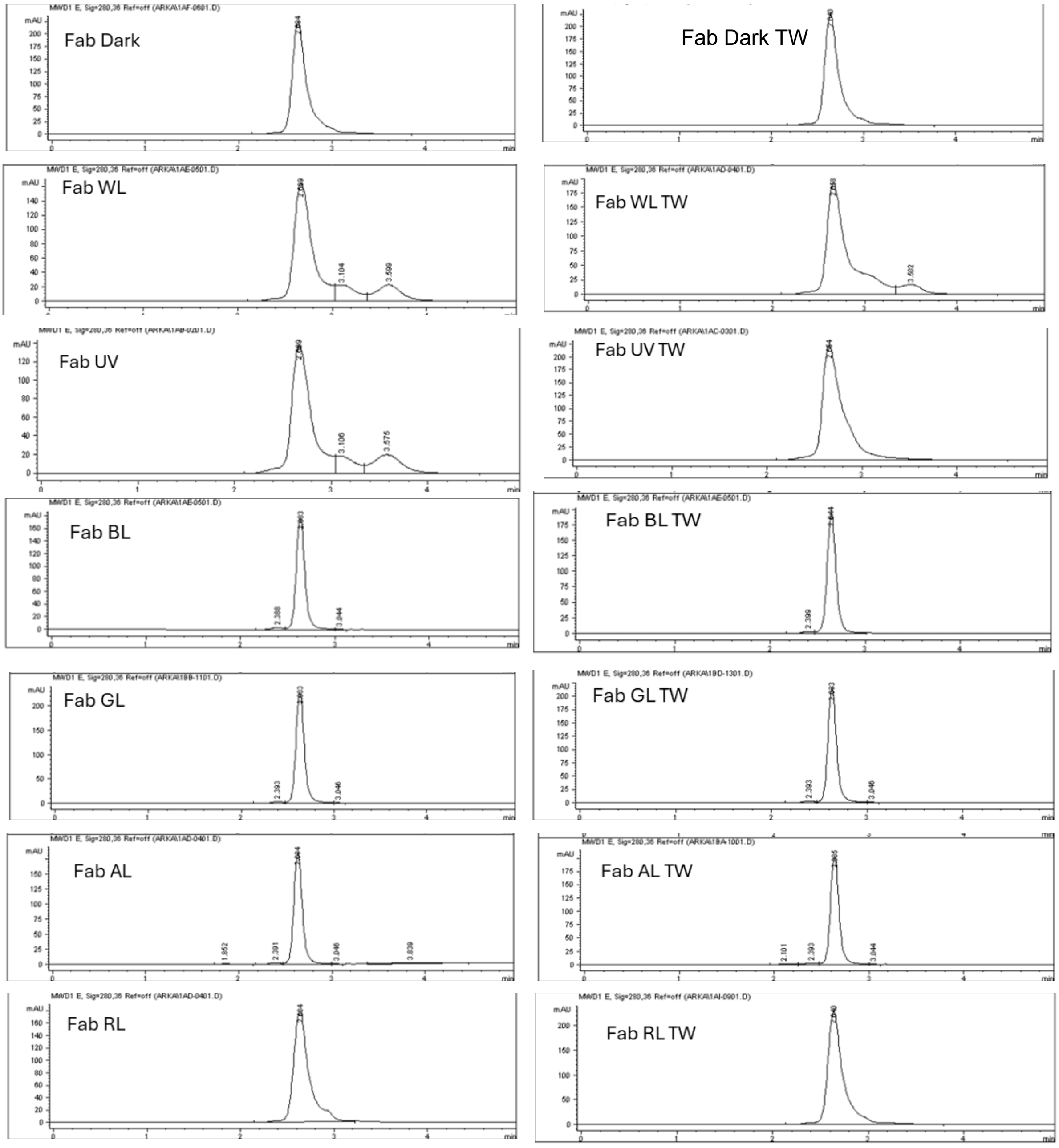

C) SEC after 30C, 600rpm, 72h, 200mM Phosphate, pH 7.5

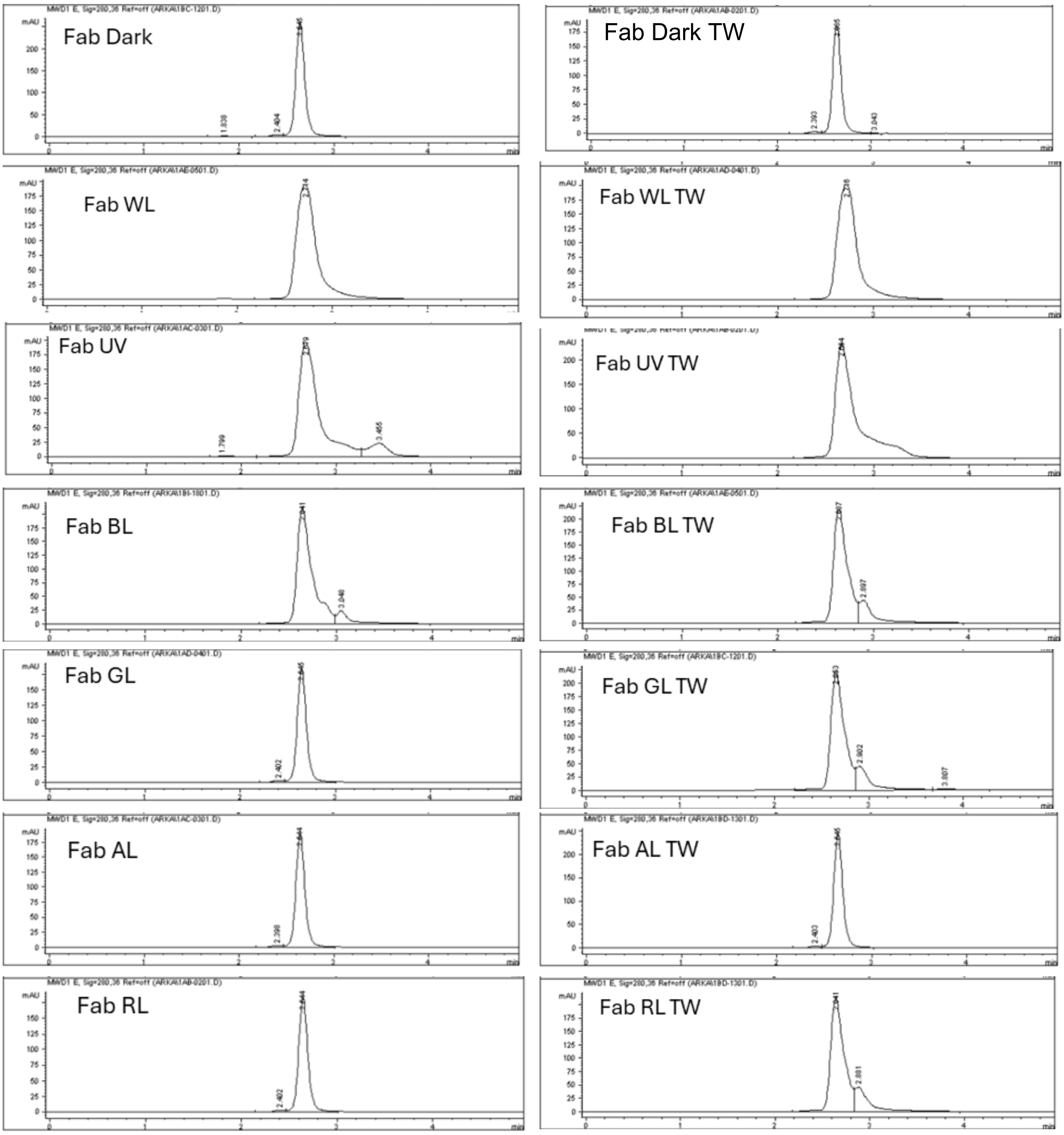

D) SEC after 21C, 600rpm, 72h, 200mM Phosphate, pH 7.5

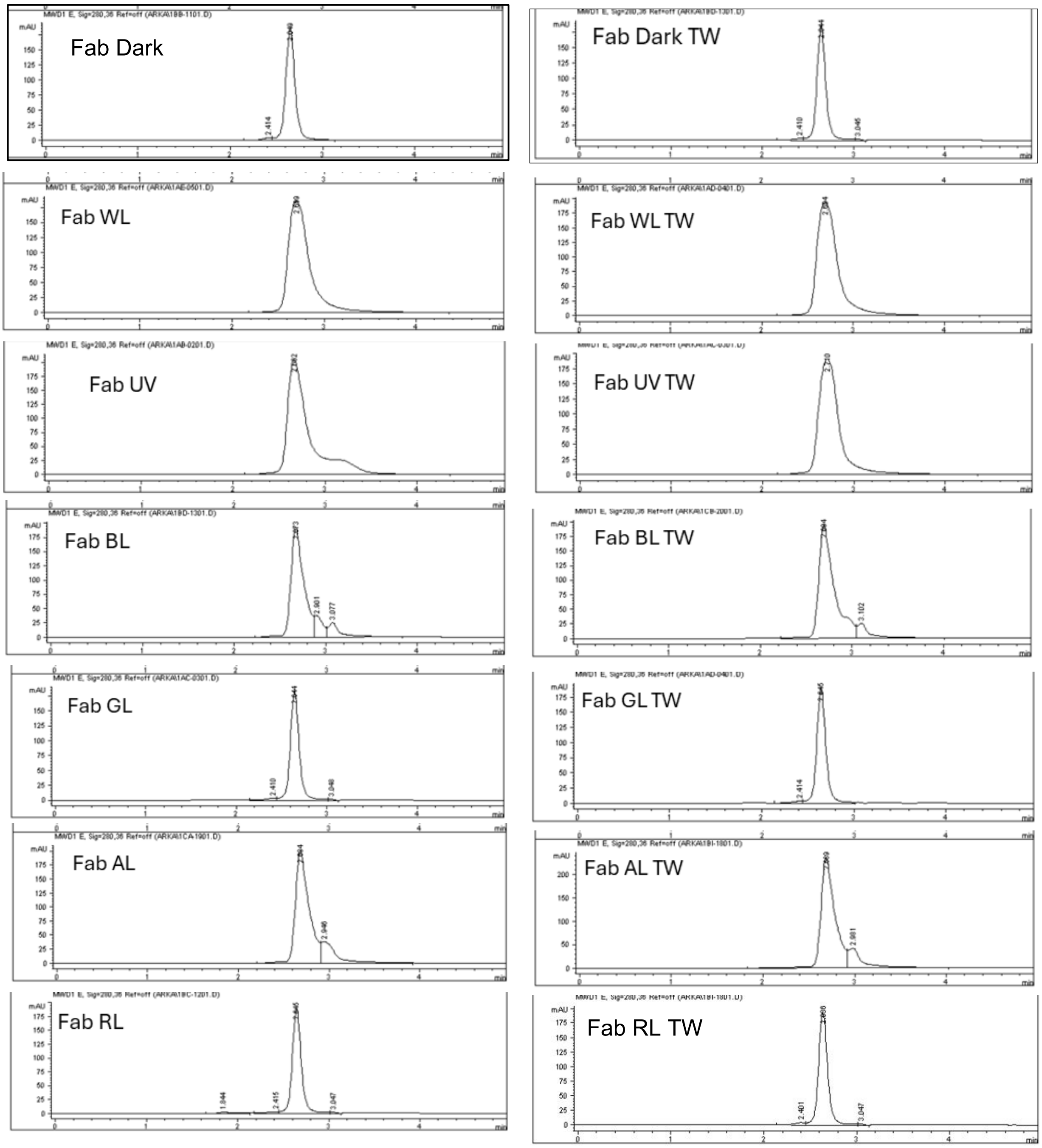

E) SEC after 10C, 600rpm, 72h, 200mM Phosphate, pH 7.5

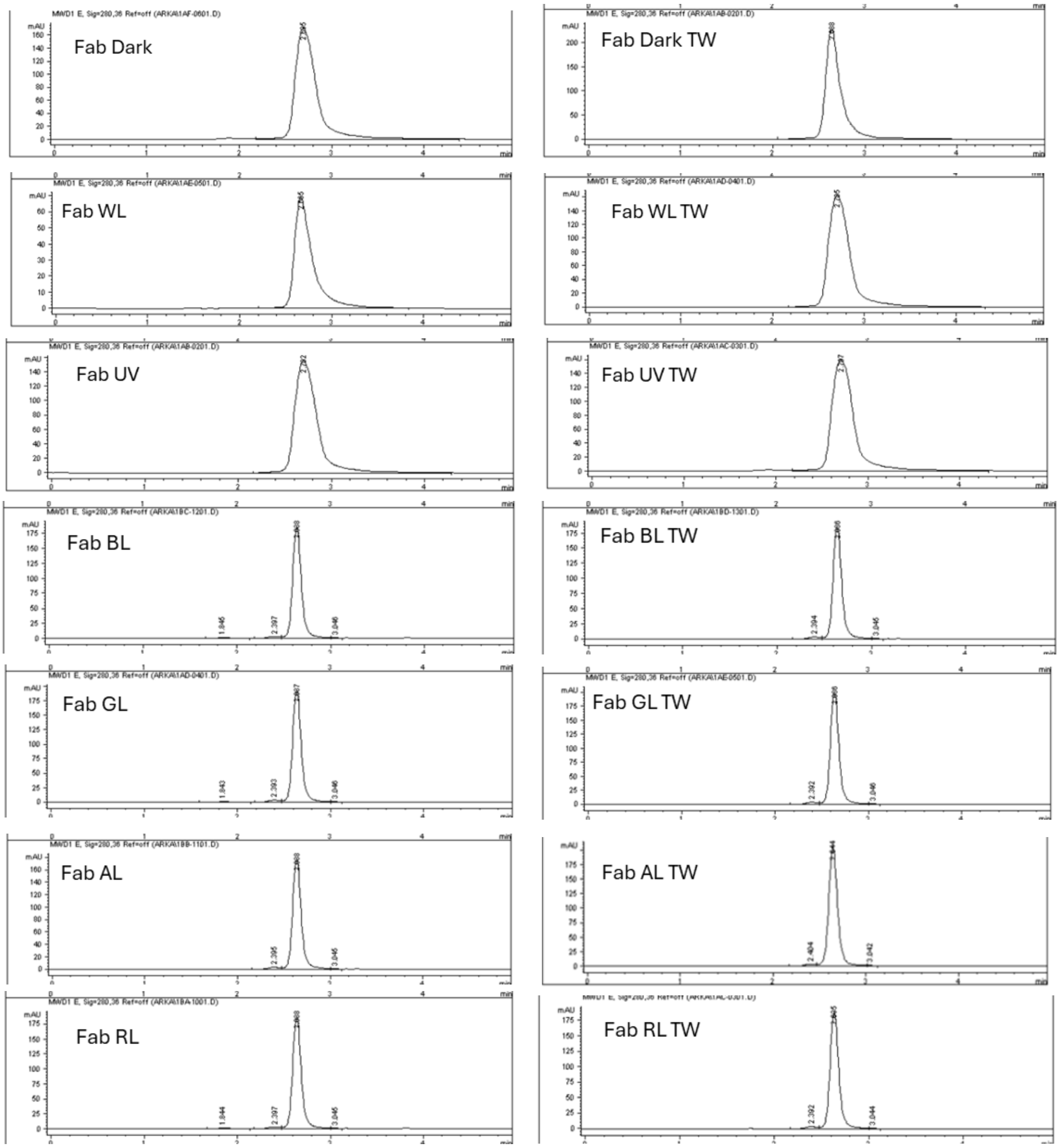

SEC after 4C, 600rpm, 72h, 200mM Phosphate, pH 7.5

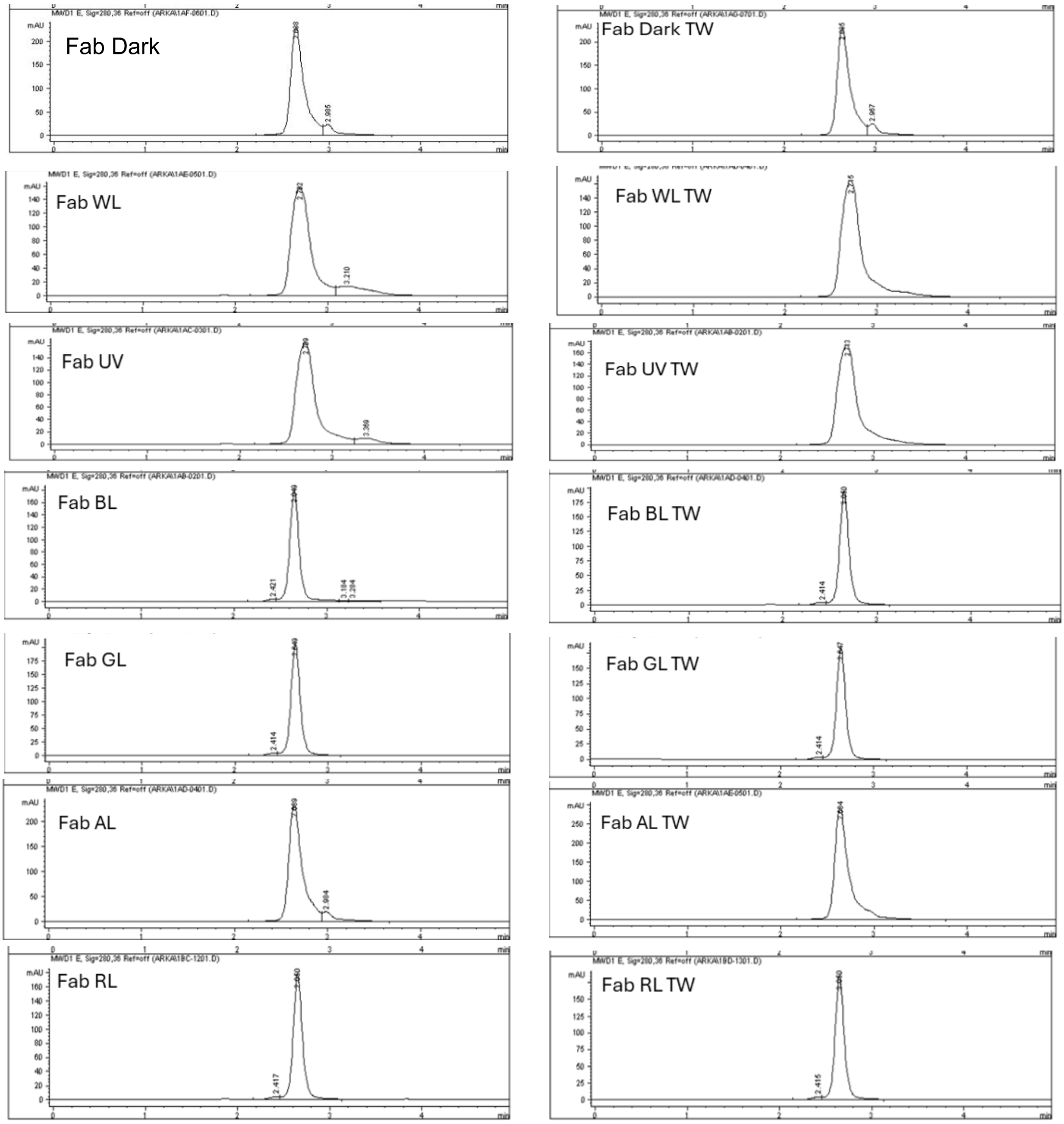

Figure S3. Comparison of monomer losses and average particle sizes (Z_{av}) as measured by SEC and DLS. 10 mg/ml Fab in phosphate buffer, pH 7.5, was incubated at 600 rpm for 72 hours in polypropylene 48-well microplates. Data are from a full factorial screen of temperature (4, 10, 21, 30, 45 °C), LED colour (blue, green, amber, red, no light), with / without plate siliconization, and the presence or absence of 0.26% (w/v) (2 mM) Tween80. A) dots coloured by light source; B) dots coloured by temperature.

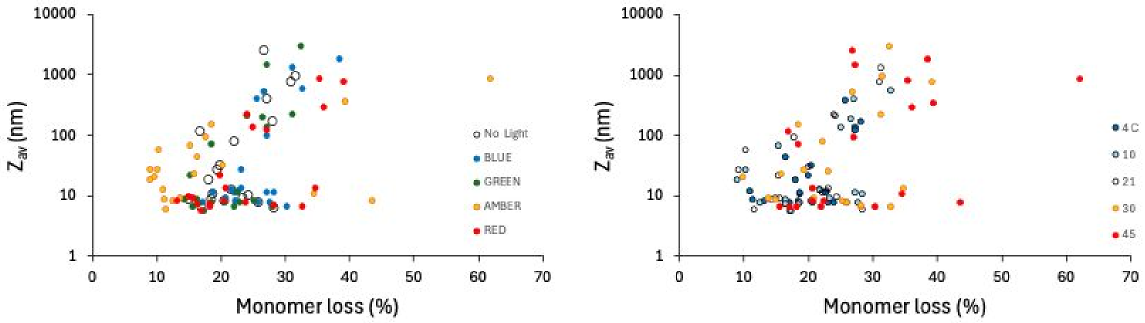

Figure S4: Deconvoluted intact mass analysis of Fab. 10 mg/ml Fab in phosphate buffer, pH 7.5, was incubated at 600 rpm for 72 hours in unsiliconised polypropylene 48-well microplates at 21 °C, under no light, or blue (BL), green (GL), UV or white (WL) LEDs, and in the presence (TW80) or absence of 0.26% (w/v) (2 mM) Tween80. The Fab control sample was not agitated and was stored in the dark at 4 °C. **A: Fab control, B: BL, TW80; C: TW80; D: GL; E: BL; F: GL, TW80; G: WL; H: WL, TW80; I: UV.**

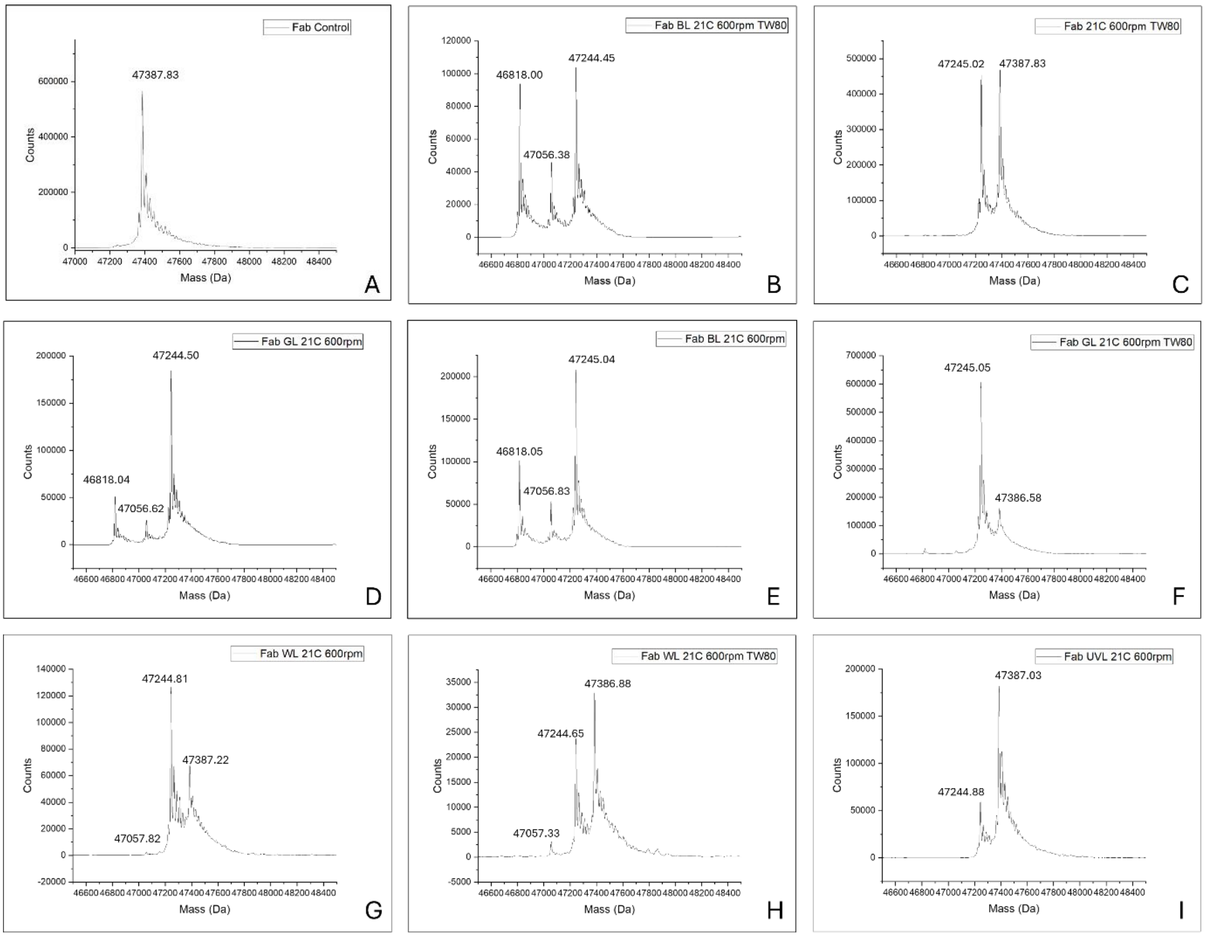

Figure S5: Reverse-phase chromatograms from LC-MS analysis. 10 mg/ml Fab in phosphate buffer, pH 7.5, was incubated at 600 rpm for 72 hours in polypropylene 48-well microplates at 21 °C, under no light, or blue (BL), green (GL), UV or white (WL) LEDs, and in the presence (T) or absence of 0.26% (w/v) (2 mM) Tween80. Plates were siliconised (S) or unsiliconised). The Fab dark control sample was not agitated and was stored in the dark at 4 °C.

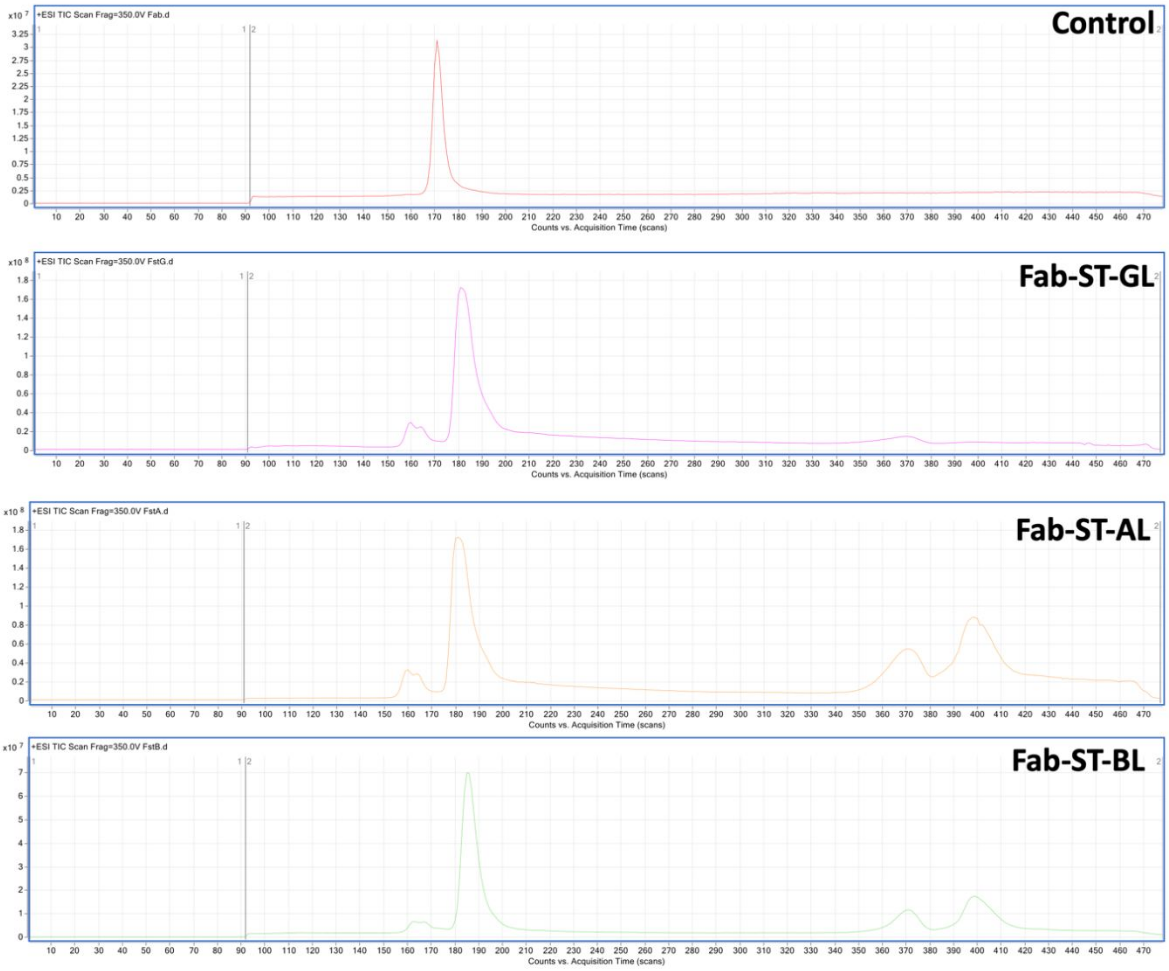

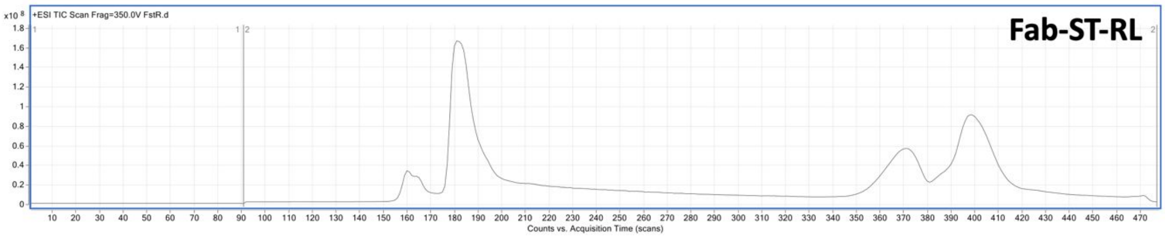
x10 8
+ESI TIC Scan Frag=350.0V FstR.d
Fab-ST-RL
Counts vs. Acquisition Time (scans)

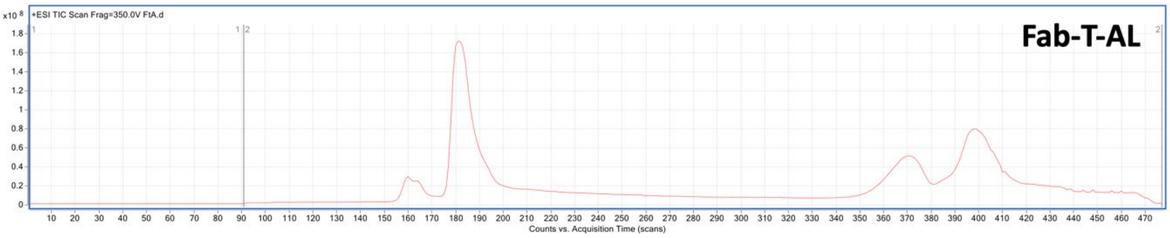
x10 8
+ESI TIC Scan Frag=350.0V FtA.d
Fab-T-AL
Counts vs. Acquisition Time (scans)

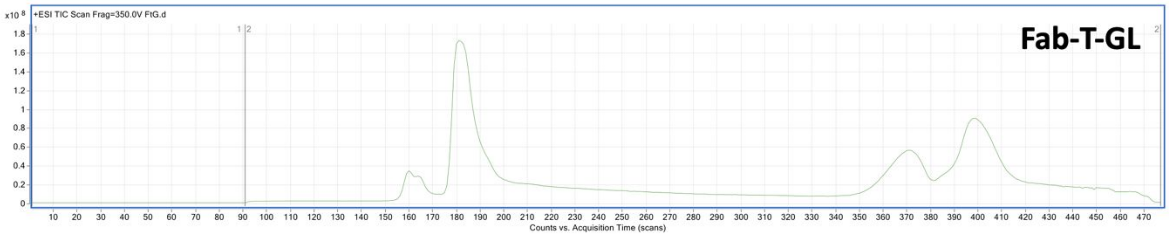
x10 8
+ESI TIC Scan Frag=350.0V FtG.d
Fab-T-GL
Counts vs. Acquisition Time (scans)

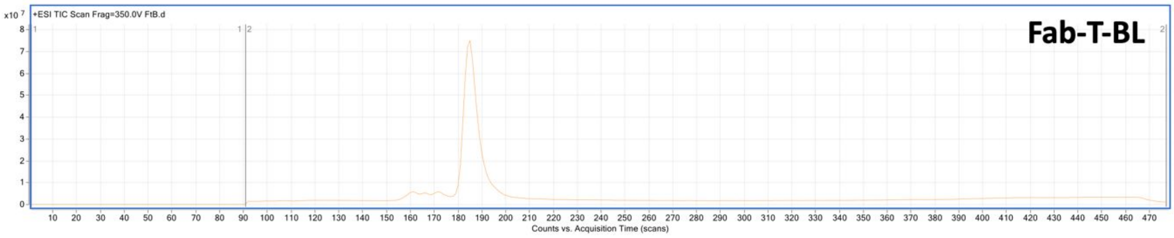
x10 7
+ESI TIC Scan Frag=350.0V FtB.d
Fab-T-BL
Counts vs. Acquisition Time (scans)

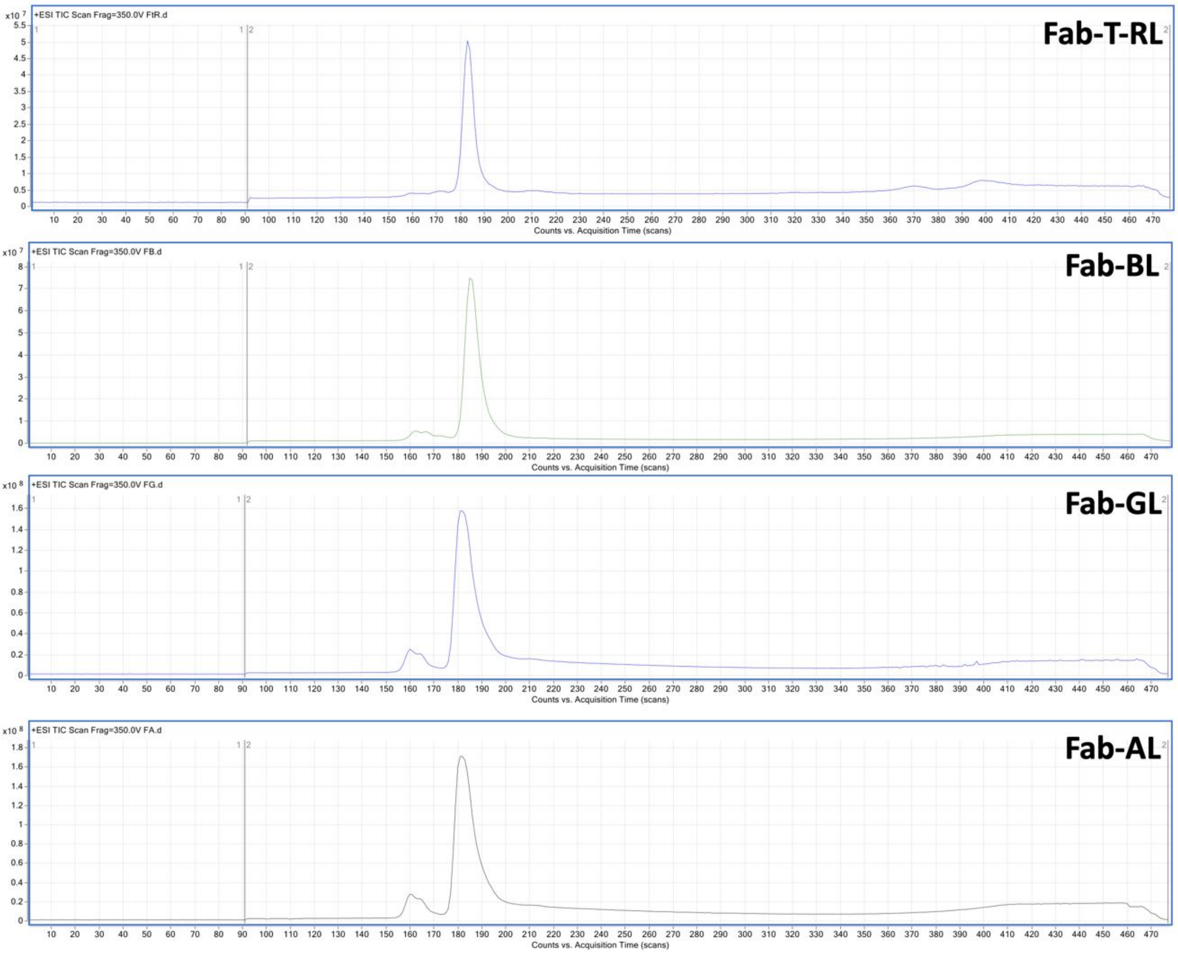
+ESI TIC Scan Frag=350.0V FtR.d
Fab-T-RL
Counts vs. Acquisition Time (scans)
+ESI TIC Scan Frag=350.0V FB.d
Fab-BL
Counts vs. Acquisition Time (scans)
+ESI TIC Scan Frag=350.0V FG.d
Fab-GL
Counts vs. Acquisition Time (scans)
+ESI TIC Scan Frag=350.0V FA.d
Fab-AL
Counts vs. Acquisition Time (scans)

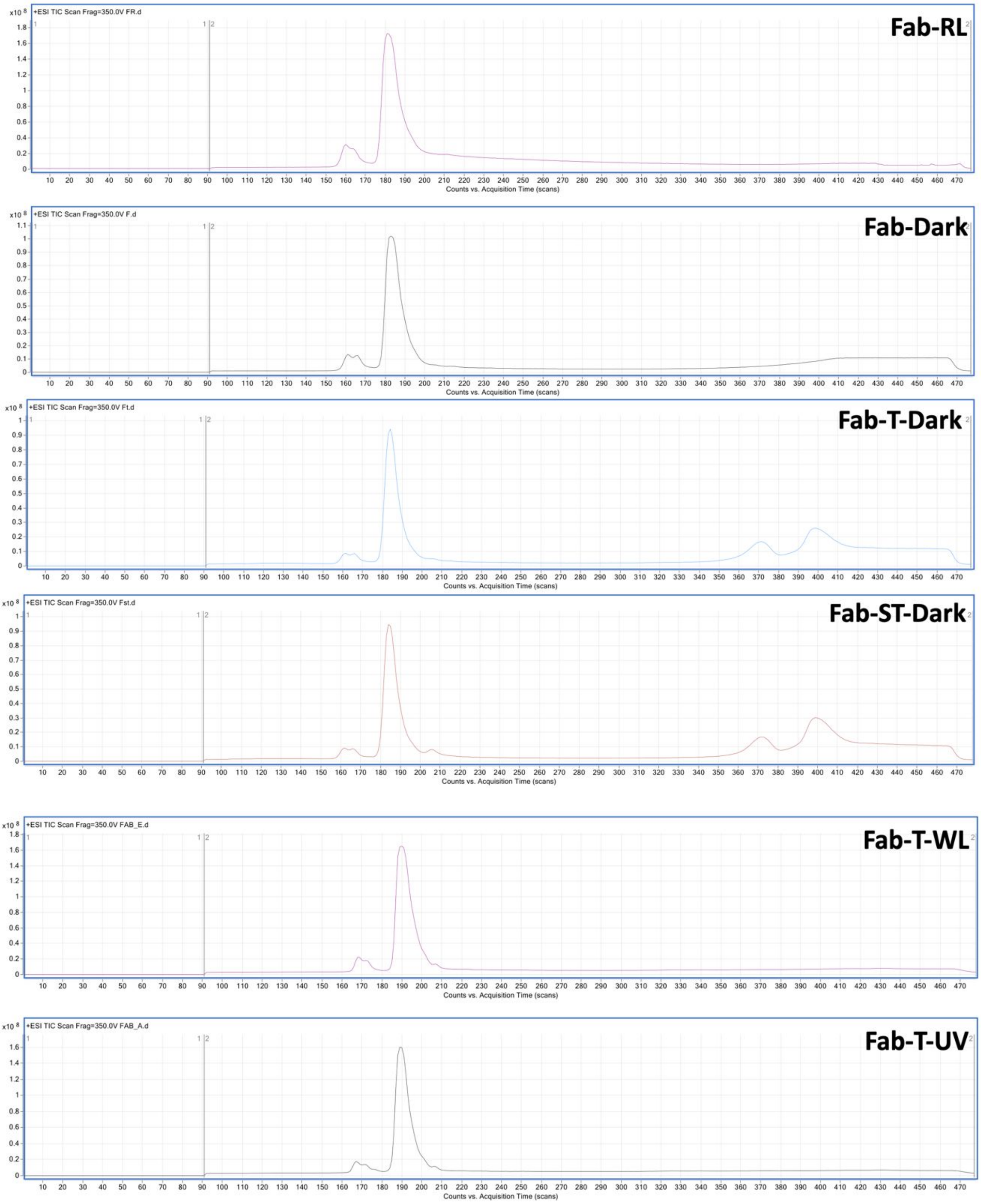
Fab-RL
+ESI TIC Scan Frag=350.0V FR.d
Fab-Dark
+ESI TIC Scan Frag=350.0V F.d
Fab-T-Dark
+ESI TIC Scan Frag=350.0V Ft.d
Fab-ST-Dark
+ESI TIC Scan Frag=350.0V Fst.d
Fab-T-WL
+ESI TIC Scan Frag=350.0V FAB_E.d
Fab-T-UV
+ESI TIC Scan Frag=350.0V FAB_A.d
x10 8
Counts vs. Acquisition Time (scans)
